# *Ehrlichia chaffeensis* TRP120 ubiquitinates tumor suppressor APC to modulate Hippo and Wnt signaling 

**DOI:** 10.3389/fcell.2024.1327418

**Published:** 2024-03-18

**Authors:** Caitlan D. Byerly, Bing Zhu, Paityn A. Warwick, LaNisha L. Patterson, Nicholas A. Pittner, Jere W. McBride

**Affiliations:** Departments of Pathology, Microbiology and Immunology, Center for Biodefense and Emerging Infectious Diseases, Sealy Institute for Vaccine Sciences and Institute for Human Infections and Immunity, University of Texas Medical Branch, Galveston, TX, United States

**Keywords:** *Ehrlichia*, ubiquitination, TRP120, adenomatous polyposis coli (APC), Hippo signaling, Wnt signaling

## Abstract

*Ehrlichia* chaffeensis: TRP120 is a multifunctional effector that acts as a ligand mimic to activate evolutionary conserved eukaryotic signaling pathways Notch, Wnt, Hedgehog and Hippo. In addition, TRP120 is also a HECT E3 ubiquitin ligase known to ubiquitinate several host cell regulatory proteins (FBW7, PCGF5 and ENO-1) for degradation. We previously determined that TRP120 ubiquitinates the Notch negative regulator, FBW7, to maintain Notch signaling and promote infection. In this study, we investigated a potential mechanism used by *Ehrlichia chaffeensis* to maintain Hippo and Wnt signaling by ubiquitinating the tumor suppressor, adenomatous polyposis coli (APC), a negative regulator of Wnt and Hippo signaling. We determined that APC was rapidly degraded during *E. chaffeensis* infection despite increased APC transcription. Moreover, RNAi knockdown of *APC* significantly increased *E. chaffeensis* infection and coincided with increased active Yap and β-catenin in the nucleus. We observed strong nuclear colocalization between TRP120 and APC in *E. chaffeensis-*infected THP-1 cells and after ectopic expression of TRP120 in HeLa cells. Additionally, TRP120 interacted with both APC full length and truncated isoforms via co-immunoprecipitation. Further, TRP120 ubiquitination of APC was demonstrated *in vitro* and confirmed by ectopic expression of a TRP120 HECT Ub ligase catalytic site mutant. This study identifies APC as a TRP120 HECT E3 Ub ligase substrate and demonstrates that TRP120 ligase activity promotes ehrlichial infection by degrading tumor suppressor APC to positively regulate Hippo and Wnt signaling.

## Introduction

*E. chaffeensis* is an obligate intracellular, Gram-negative bacterium and the etiologic agent of the prevalent and life-threatening zoonosis, human monocytotropic ehrlichiosis (HME) (Paddock et al., 1997; Paddock and Childs, 2003). *E. chaffeensis* exhibits tropism for mononuclear phagocytes and activates conserved cellular signaling pathways via tandem repeat protein (TRP) effectors to promote intracellular survival (Rogan et al., 2021; Byerly et al., 2022; Patterson et al., 2022; Byerly et al., 2023a). TRP effectors are multifunctional proteins secreted by the type-1 secretion system that interact with a diverse group of host cell proteins involved in essential cellular processes (Wakeel et al., 2009; Luo et al., 2011; Wakeel et al., 2011; Luo and McBride, 2012; Lina et al., 2016; Luo et al., 2017; Luo et al., 2018; Byerly et al., 2021). Notably, the 120 kDa tandem repeat protein (TRP120) has emerged as a model moonlighting effector that functions as a cell signaling pathway ligand mimic, ubiquitin ligase and nucleomodulin. More recently, TRP120 has been shown to activate evolutionarily conserved signaling pathways such as Hippo, Wnt, Notch, and Hedgehog through short linear motif (SLiM) ligand mimicry (Rogan et al., 2021; Byerly et al., 2022; Patterson et al., 2022; Byerly et al., 2023b; Rogan et al., 2021; Byerly et al., 2022; Patterson et al., 2022; Byerly et al., 2023a).

TRP120 contains a novel Wnt SLiM within each tandem repeat that interacts with Wnt Fzd receptors to activate Hippo and Wnt transcription factors, Yap and β-catenin, to inhibit intrinsic host cell apoptosis (Rogan et al., 2021; Byerly et al., 2023a). Hippo and Wnt signaling are regulated by the tumor suppressor, adenomatous polyposis coli (APC), which targets and shuttles Yap and β-catenin out of the nucleus to a destruction complex within the cytoplasm (Henderson, 2000; Azzolin et al., 2014; Cai et al., 2015). The functionality of APC differs between the full length (FL) and truncated (TR) isoforms, which have been tied to various roles during Hippo and Wnt signaling. Both isoforms are known to shuttle between the cytoplasm and nucleus using nuclear export sequences (Brocardo et al., 2005). APC-FL chaperones Yap and β-catenin between the nucleus and cytoplasm for proteolysis to negatively regulate transcriptional activation (Henderson, 2000; Cai et al., 2015). Conversely, APC-TR does not act as a chaperone, but binds β-catenin to downregulate β-catenin gene target expression (Schneikert et al., 2007). Thus, APC-FL and -TR forms are relevant in preventing Yap and β-catenin transcriptional activation. Further, MKRN1 is an E3 ligase known to ubiquitinate and degrade APC to enhance Wnt signaling, demonstrating that APC is regulated by ubiquitin and a potential substrate exploited by pathogens (Lee et al., 2018).

*E. chaffeensis* TRP120 has well described HECT E3 ligase activity and several host cell substrates have been reported including PCGF5, FBW7 and ENO-1, which are involved in chromatin remodeling, regulation of Notch signaling and glycolytic flux, respectively (Zhu et al., 2017; Mitra et al., 2018; Wang et al., 2020; Zhu and McBride, 2021). Previous studies have shown that *E. chaffeensis* TRP120 ubiquitination and degradation of FBW7, the substrate recognition unit of the Skp-1-cullin-1-FBOX E3 Ub ligase, increases levels of Notch and other oncoproteins to promote infection. Other Gram-negative bacteria possess E3 ubiquitin ligases, including *Salmonella, Shigella, Sinorhizobium,* and *Ralstonia* (Kim et al., 2005; Zhang et al., 2006; Diao et al., 2008; Ashida et al., 2013; Bullones-Bolanos et al., 2022). *Salmonella enterica* utilizes ubiquitin ligase effectors including the HECT-like E3 ligase, SopA. SopA ubiquitinates host RING E3 ubiquitin ligases, TRIM56 and TRIM65, to modulate type I interferon signaling and inflammation (Kamanova et al., 2016). In addition, *Shigella flexneri* secretes several NEL E3 ligase effectors that target and downregulate NF-κB signaling (Ashida et al., 2013).

Considering *E*. *chaffeensis* TRP120 is known to exploit Hippo and Wnt signaling (Rogan et al., 2021; Byerly et al., 2023b), we examined the relationship between TRP120 HECT E3 ligase activity and the tumor suppressor APC in modulating Hippo and Wnt transcriptional activators, Yap and β-catenin. We determined that *E. chaffeensis* TRP120 promotes intracellular infection by ubiquitinating APC for degradation to maintain nuclear Yap and β-catenin levels. Although we previously demonstrated activation of Yap and β-catenin via TRP120 Wnt ligand mimicry, TRP120 ubiquitination of APC is an additional mechanism employed by *E*. *chaffeensis* to prevent the negative regulation of Yap and β-catenin and secure downstream effects of Hippo and Wnt signaling for intracellular survival.

## Results

### APC is degraded during *Ehrlichia chaffeensis* infection

**FIGURE 1:**
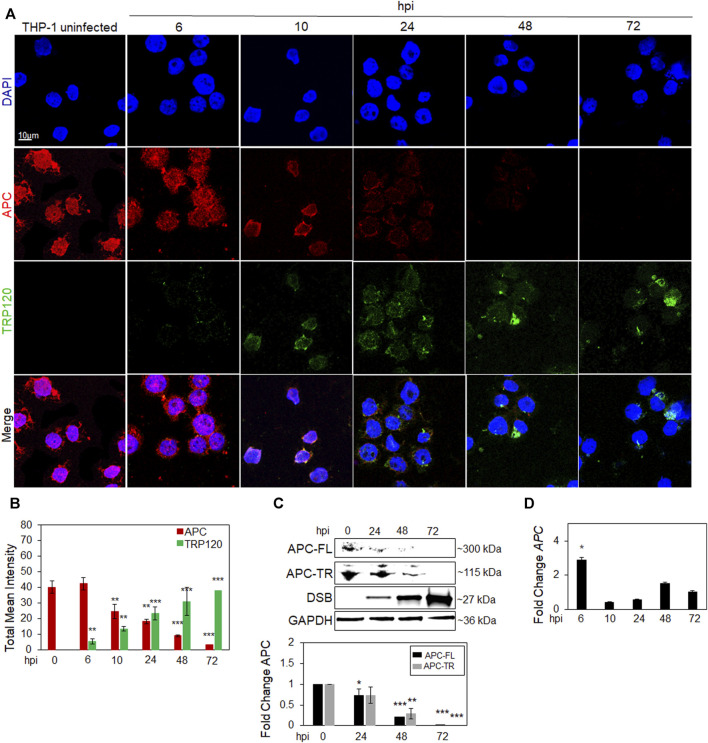
APC is degraded during *E. chaffeensis* infection. **(A)** Confocal immunofluorescent microscopy was performed to observe temporal changes of total APC overtime (red) during *E. chaffeensis* infection represented by TRP120 (green). *E. chaffeensis*-infected THP-1 cells were harvested at 0, 6, 10, 24, 48 and 72 h post-infection (hpi) (scale bar = 10 μm). **(B)** Intensity graphs demonstrate the total mean intensity of APC and TRP120 in THP-1 cells selected from randomized areas/slide (n = 10). Analysis was performed using ImageJ to determine mean grey value from randomized areas/slide (n = 10) and data shown as mean ± SD (*p < 0.05; **p < 0.01). **(C)** Western blot analysis depicting APC full length (FL) and truncated (TR) levels at 0, 24, 48 and 72 hpi with GAPDH as a loading control. Anti-Dsb antibody was used to detect *E. chaffeensis* infection. Bar graph (below) represents densitometry values of Western immunoblot normalized to GAPDH. The fold change of APC is relative to uninfected control and significance determined using a pairwise *t*-test. **(D)** Table represents expression of *APC* gene in *E. chaffeensis-*infected cells normalized to uninfected cells, with no significant downregulation detected. **(C–D)** Experiments were performed with three biological and technical replicates for *t*-test analysis. Data are represented as means ± SD (*p < 0.05; **p < 0.01; ***p < 0.001).

We previously demonstrated the activation of Yap and β-catenin via TRP120 Wnt ligand mimicry (Rogan et al., 2021; Byerly et al., 2023b). However, multiple host cellular proteins are involved in Yap and β-catenin negative regulation, including APC. Since TRP120 is a known HECT E3 Ub ligase, we investigated whether APC is degraded during infection in *E. chaffeensis* infected THP-1 cells (Figure 1). Confocal immunofluorescent micrographs demonstrate temporal degradation of APC and an increase in TRP120 during infection (Figures 1A,B). Moreover, a progressive nuclear reduction in APC level was observed. We confirmed the results using Western blot and PCR to determine APC protein and gene expression levels. Interestingly, APC protein levels significantly decrease overtime, but *APC* gene transcription did not, indicating that APC is degraded during infection (Figures 1C,D).

**FIGURE 2:**
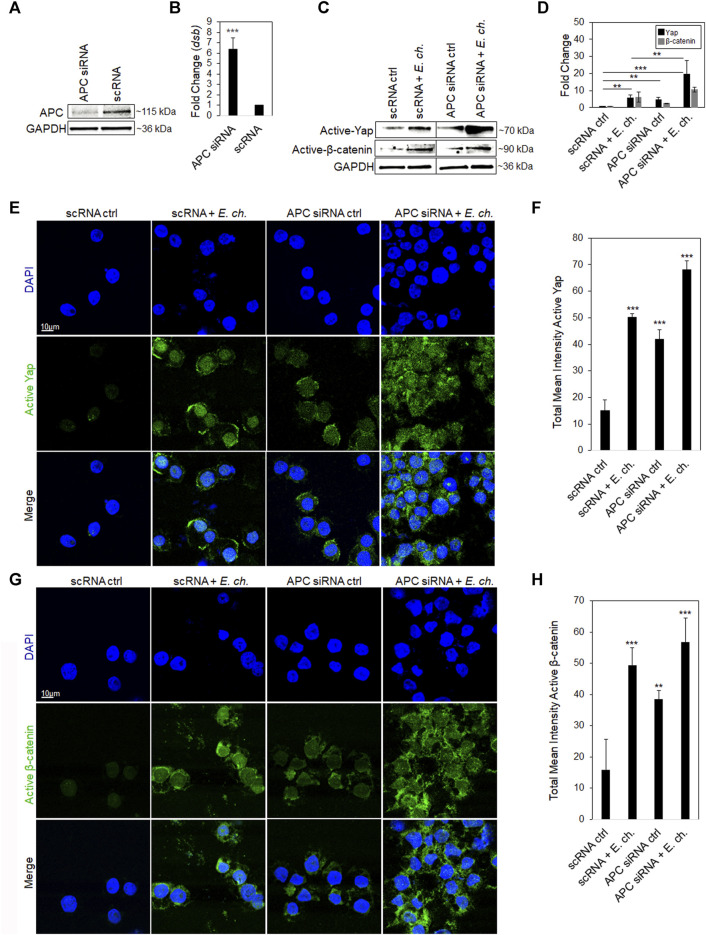
APC knockdown stabilizes active Yap and β-catenin levels in the nucleus during *E. chaffeensis* infection. **(A)** Western immunoblots showing APC knockdown efficiency using small interfering RNA-transfected (siRNA) THP-1 cell whole-cell lysates, with scrambled siRNA (scrRNA) transfected as control (24 hpt). siRNA knockdown (%) indicates total percent knockdown of APC relative to control and normalized to GAPDH. **(B)** THP-cells (24 hpt) were infected with *E. chaffeensis* (MOI 100) and harvested 24 hpi. *E. chaffeensis*infected scrRNA cells represent the positive control. qPCR amplification of the ehrlichial disulfide bond formation protein (*dsb*) gene was used to quantify *E. chaffeensis* infection. APC knockdown was performed with three biological and technical replicates for *t*-test analysis. **(C)** Western blot analysis depicting active Yap and β-catenin for *E. chaffeensis*-infected or uninfected APC knockdown cells relative to scrambled control. **(D)** Bar graph represents densitometry values of Western immunoblot normalized to GAPDH. **(E,G)** *E. chaffeensis* infected or uninfected APC knockdown cells were analyzed using confocal immunofluorescent microscopy to observe active Yap and β-catenin relative to control. Randomized areas/slide (n = 10) were selected to quantitate active Yap and β-catenin. **(F,H)** Intensity graphs demonstrate the total mean intensity of active Yap and β-catenin. Analysis was performed using ImageJ to determine mean grey value from randomized areas/slide (n = 10). Experiments were performed with three biological and technical replicates for *t*-test analysis. Data are represented as means ± SD (*p < 0.05; **p < 0.01; ***p < 0.001). Further, statistical analysis using ANOVA indicates significant differences in the expression of both Yap and β-catenin among the groups (*p* < 0.05). Post-hoc Tukey tests was used to determine statistically significant differences between the groups.

**APC knockdown stabilizes active Yap and β-catenin levels in the nucleus during *E. chaffeensis* infection.** *E. chaffeensis* is a known Hippo and Wnt modulator that activates Yap and β-catenin transcriptional regulators for intracellular survival (Rogan et al., 2021; Byerly et al., 2023b). Since APC negatively regulates active Yap and β-catenin by shuttling them out of the nucleus, we examined the effect of APC on *E. chaffeensis* infection using RNAi to knockdown APC in THP-1 cells. *E. chaffeensis* infection increased ∼7-fold in APC knockdown (KD) cells compared to the scrambled siRNA (scRNA) control (Figure 2A). Additionally, we examined whether APC KD influenced Yap and β-catenin during *E. chaffeensis* infection. Both activated Yap and β-catenin levels in the nucleus significantly increased in *E. chaffeensis*-infected APC KD cells compared to controls (uninfected APC KD cells and *E. chaffeensis*-infected and uninfected scRNA transfected cells), demonstrating that APC stabilizes activated Yap and β-catenin levels in the nucleus during infection (Figures 2B,C). Further, to examine the cellular distribution of Yap and β-catenin levels in *E. chaffeensis*-infected APC KD cells compared to controls, cells were stained with anti-active Yap and β-catenin antibodies and examined by confocal microscopy. A significant increase in activated Yap and β-catenin was observed within the nucleus of *E. chaffeensis*-infected APC KD cells compared to controls, demonstrating that APC negatively regulates Yap and β-catenin levels in the nucleus during infection (Figure 2D,E).

### *Ehrlichia chaffeensis* TRP120 nuclear colocalization and interaction with APC

**FIGURE 3:**
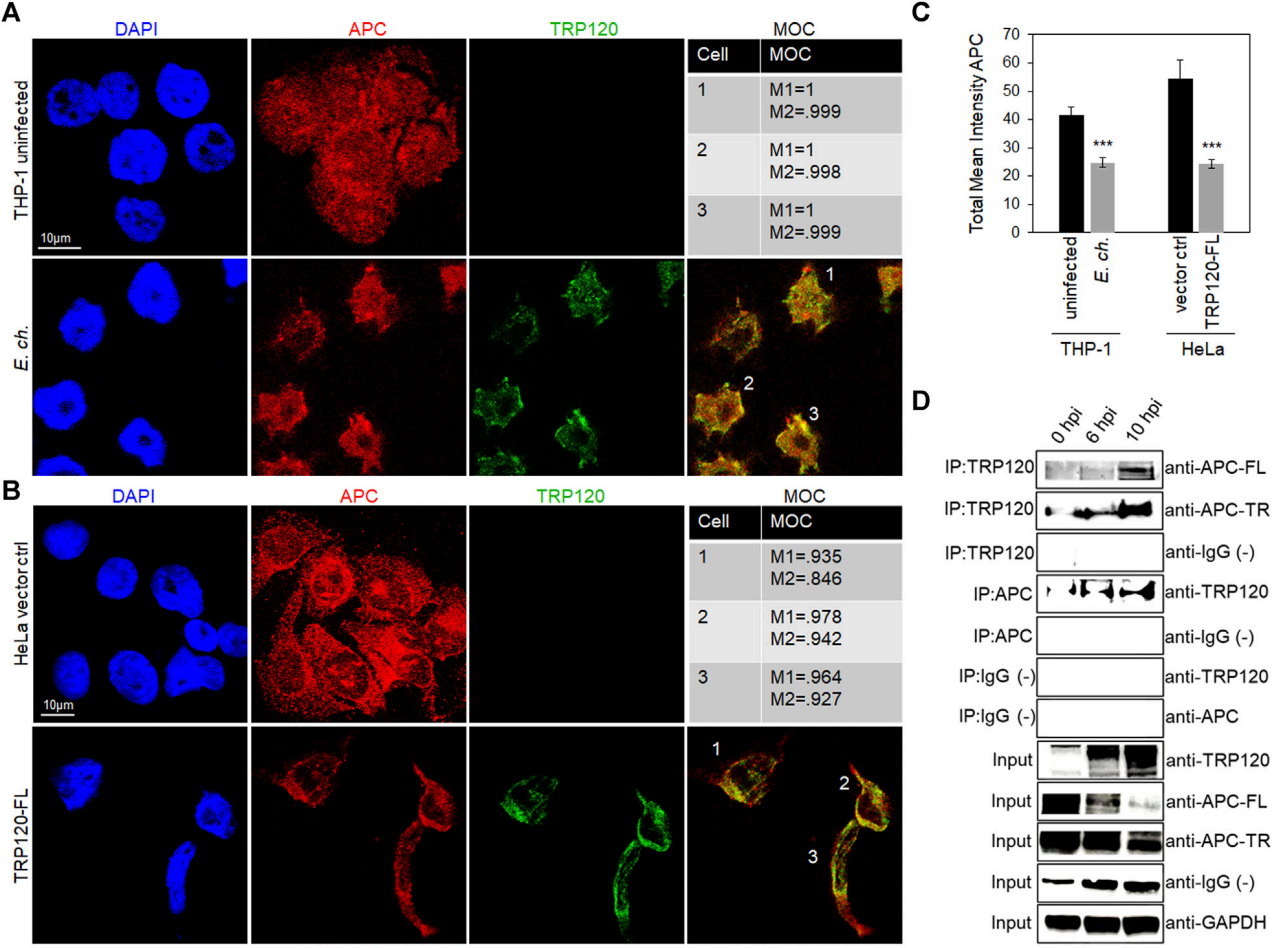
*E*. *chaffeensis* TRP120-APC nuclear colocalization and interaction with APC full length and truncated forms. **(A)** Confocal microscopy demonstrating the distribution of TRP120 and total APC in uninfected and *E. chaffeensis*-infected THP-1 cells (MOI 100). Co-localization of TRP120 (green) with total APC (red) 10 hpi compared to uninfected cells. **(B)** HeLa cells were transfected with TRP120-FL to examine colocalization of TRP120 (green) with APC (red) by confocal microscopy. **(A, B)** Experiments were performed with at least three biological and technical replicates. Randomized areas/slide (n = 10) were used to detect interaction. Mander’s overlap coefficient (MOC) indicates a correlation between TRP120 and total APC, suggesting a direct interaction. (scale bar = 10 μm). **(C)** Intensity graphs demonstrate the total mean intensity of APC in respective THP-1 and HeLa cell samples. Analysis was performed using ImageJ to determine mean grey value from randomized areas/slide (n = 10) and data is shown as mean ± SD (***p < 0.001) **(D)** Co-immunoprecipitation (Co-IP) and reverse Co-IP confirm a direct interaction between TRP120 with APC FL and TR forms. THP-1 cells were infected with cell-free *E*. *chaffeensis* and then harvested at 0, 6, and 10 hpi. Western blot analysis was normalized to GAPDH expression and experiment was repeated with three biological replicates.

*E. chaffeensis* TRP120 is known to interact with and ubiquitinate various host proteins, including PCGF5, FBW7 and ENO-1. To determine whether TRP120 interacts with APC, we visualized the cellular distribution and colocalization of APC with TRP120-expressing ehrlichial inclusions. *E. chaffeensis-*infected THP-1 cells were stained with anti-APC and anti-TRP120 antibodies, mounted with DAPI, and examined by confocal microscopy. We found distribution of APC within the cytoplasm and nucleus in uninfected THP-1 cells. However, in *E. chaffeensis-*infected cells, we found colocalization of APC with TRP120 within the nucleus at 10 hpi (Figure 3A). In addition, colocalization of native APC with ectopically expressed TRP120-FL was also observed in transfected HeLa cells (Figure 3B). Intensity correlation analysis using ImageJ demonstrated a positive Mander’s overlap coefficient (MOC) between APC and TRP120 within the cytoplasm and nucleus (Figures 3A,B). Further, total mean intensity of APC was calculated using ImageJ. There was significantly less APC in *E. chaffeensis*-infected cells and TRP120-FL ectopically expressed cells compared to respective controls, suggesting APC degradation (Figure 3C). Since colocalization only infers a direct interaction between TRP120 and APC, we performed co-immunoprecipitation (Co-IP) to confirm a direct interaction. TRP120 or APC (reverse Co-IP) immunoprecipitates from lysates of *E. chaffeensis-*infected THP-1 cells harvested at 0 (uninfected control) 6 and 10 hpi, demonstrating a direct interaction between TRP120 and APC. We found high levels of APC-TR and -FL bound to TRP120 in the infected THP-1 cells compared to uninfected controls. (Figure 3D).

**FIGURE 4:**
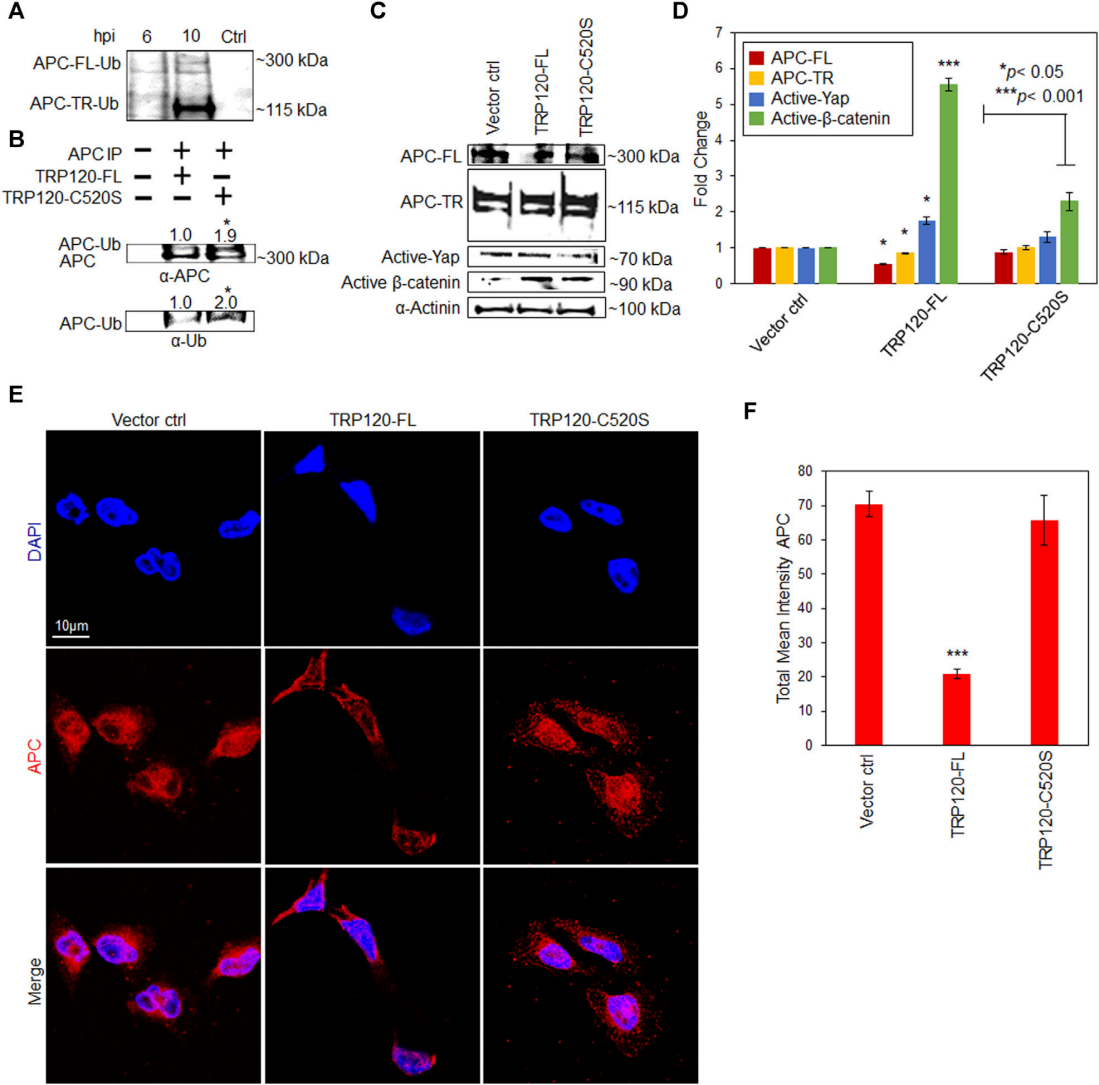
*E. chaffeensis* TRP120 HECT E3 Ub ligase ubiquitinates APC to modulate active Yap and β-catenin levels in the nucleus. **(A)** Ubiquitination of native APC in *Ehrlichia chaffeensis*-infected THP-1 cell lysates harvested at 6 and 10 hpi compared to uninfected control. Lysates were subjected to ubiquitin pulldowns using a Ubiquitin Enrichment Kit. Ubiquitinated APC was detected by Western blot. **(B)** Western immunoblot and densitometry (fold change, mean of three replicates) of *in vitro* ubiquitination of APC (APC-Ub) incubated with rTRP120-FL or rTRP120-C520S and probed with anti-APC and Ub antibodies. **(C)** HeLa cells transfected with TRP120-FL or catalytically inactive TRP120 C520S Ub mutant for 24 h and collected for Western blot analysis of APC, active Yap and β-catenin. APC protein expression is significantly higher in the presence of TRP120-C520S while active Yap and β-catenin are significantly lower, suggesting that TRP120 ubiquitinates APC to stabilize Yap and β-catenin levels in the nucleus. **(D)** Bar graph represents densitometry values of Western blot normalized to GAPDH. **(E)** Confocal microscopy demonstrating the distribution of total APC in HeLa cells transfected with TRP120-FL or TRP120 C520S compared to vector control. Randomized areas/slide (n = 10) were used to detect APC (scale bar = 10 μm). **(F)** Intensity graphs demonstrate the total mean intensity of APC in HeLa cells. Experiments were performed with three biological and technical replicates for *t*-test. Data are represented as means ± SD (*p < 0.05; ***p < 0.001). ANOVA was used to determine significant differences among APC-FL, APC-TR. activated Yap, and activated β-catenin groups (*p* < 0.05). Post-hoc Tukey tests was used to identify statistically significant differences between the groups.

***E. chaffeensis* TRP120 HECT E3 Ub ligase ubiquitinates APC to modulate Yap and β-catenin.** TRP120 HECT E3 ligase targets various host cell proteins involved in chromatin remodeling, cell signaling, and glycolytic flux. To determine if APC is a substrate of TRP120 Ub ligase activity we examined APC ubiquitination levels during infection. Uninfected and *E. chaffeensis*-infected cell lysates were subjected to ubiquitin enrichment, and APC-TR-Ub and APC-FL-Ub were identified at 6 and 10 hpi compared to uninfected controls (Figure 4A). *In vitro* ubiquitination assay of IP-purified native APC with rTRP120-FL or rTRP120-C520S detected ubiquitination of APC in the presence of rTRP120-FL compared to catalytically inactive rTRP120-C520S (Figure 4B). The results demonstrate that TRP120 directly targets APC for ubiquitination during infection. Further, we transfected HeLa cells with either TRP120-FL or TRP120 E3 Ub ligase mutant (TRP120-C520S) and determined by immunoblot and confocal microscopy that TRP120 utilizes HECT E3 Ub ligase function to negatively regulate APC (Figures 4C–F) and stabilize active Yap and β-catenin in the nucleus (Figures 4C,D).

## Methods

### Cell culture and *Ehrlichia chaffeensis* cultivation

Human monocytic leukemia cells (THP-1; ATCC TIB-202) were propagated in RPMI 1640 with L-glutamine and 25 mM HEPES buffer (Invitrogen, Carlsbad, CA), supplemented with 10% fetal bovine serum, and incubated at 37°C in a 5% CO_2_ atmosphere. Henrietta Lack’s cervical epithelial adenocarcinoma (HeLa) cells were propagated in MEM medium with Earle’s Salts and L-glutamine (Thermo Fisher Scientific, Waltham, MA), supplemented with 10% fetal bovine serum. *E*. *chaffeensis* (Arkansas strain) was cultivated in THP-1 cells as previously described (Byerly et al., 2022).

### RNAi and *Ehrlichia* quantification

APC ON-TARGETplus siRNA (Dharmacon, Lafayette, Co) was used to perform APC knockdown as previously described (Rogan et al., 2021; Byerly et al., 2022). Scrambled RNAi was used as siRNA control. THP-1 cells were infected with cell-free *E. chaffeensis* (MOI 100) 24 h post-transfection. Cells were harvested at 24 hpi and ehrlichial load was determined using qRT-PCR as described below. All knockdowns were performed with three biological and technical replicates and significance was determined using a *t*-test analysis.

### Quantitative real time PCR

Total RNA was isolated from each sample (1 × 10^6^ cells/sample) using RNeasy Mini kit (Qiagen, Hilden, Germany). On column, DNA digestion was performed using the RNase-free DNase kit (Qiagen). The concentration and the purity of RNA were determined using a Nanodrop 100 spectrophotometer (Thermo Fisher). cDNA was synthesized from total RNA (1.0 μg) using iScript cDNA Synthesis Kit (BioRad, Hercules, CA), according to the manufacturer’s protocol. Additionally, SideStep Lysis & Stabilization Buffer (Agilent) was used for siRNA experiments to lyse THP-1 cells and stabilize the release of nucleic acids. qRT-PCR was performed using RT2 Profiler PCR array in combination with RT2 SYBR green master mix (Qiagen) using a QuantStudio 6 Flex real-time PCR system (Thermo Fisher Scientific). PCR conditions and analysis were conducted as previously described (Byerly et al., 2022).

PCR primer sequences included *APC* (F: 5′-GAC​TCG​GAA​ATG​GGG​TCC​AA-3’; R: 5′-GGT​GCA​GAG​TGT​GTG​CTA​CT-3′ used for experiment in Figure 1 *dsb* (F: 5′-GCT​GCT​CCA​CCA​ATA​AAT​GTA​TCC​T-3’; R: 5′-GTT​TCA​TTA​GCC​AAG​AAT​TCC​GAC​ACT-3′) used for experiment in Figure 2. Relative gene expression was calculated by determining the cycle threshold (Ct) value and normalizing to *GAPDH*.

### Western immunoblot

Briefly, THP-1 cells (100% confluent) were harvested, and lysates prepared using RIPA buffer (Sigma-Aldrich, Burlington, MA) supplemented with complete mini EDTA-free protease inhibitor (Roche, Basel, Switzerland) and phenylmethene-sulfonylfluoride PMSF (10 mM) (Sigma-Aldrich). Cell lysate protein concentrations were determined and Western blots were performed as previously described (Byerly et al., 2022) using α-APC (1:500) (Abcam, Cambridge, United Kingdom, ab40778), α-active Yap (Abcam, ab205270) and α-non-phospho (active) β-catenin (1:200) (Cell Signaling, Danvers, MA, 4270S).

### Confocal microscopy

Cells were prepared for confocal microscopy as previously described and stained with monoclonal antibodies described above; mouse α-APC (1:100), rabbit α-active Yap (1:200), rabbit α-non phospho (Active) β-catenin (1:100), rabbit α-TRP120-I1 (1:750) and rabbit α-Dsb (1:500). Secondary antibodies were α-rabbit IgG (H + L) Alexa Fluor Plus 594 and α-mouse or rabbit IgG (H + L) Alexa Fluor Plus 488 (1:200) (Invitrogen). Zeiss LSM 880 laser confocal microscope was utilized for all confocal micrographs and analyzed with Zen black and Fiji software. Randomized areas/slide (n = 10) were used to detect proteins of interest. Experiments were performed with three biological and technical replicates.

### Transfection

HeLa cells (1×10^6^) were seeded in a 60 mm culture dish 24 h prior to transfection. All proteins were expressed in a pcDNA3.1+C-6His vector. TRP120 full-length (pcDNA3.1+TRP120_FL_C-6His) and the HECT Ub ligase catalytic inactive mutant (pcDNA3.1+TRP120_C520S_C-6His) were cloned into the pcDNA3.1+C-6His vector at NheI/XbaI sites. pcDNA3.1+C-6His empty vector was used as a control. All vectors were added to Opti-MEM and Lipofectamine 3,000 mixture and incubated for 20 min at 37°C. Lipofectamine/plasmid mixtures were added to HeLa cells and incubated for 4 h at 37°C. The medium was aspirated 4 h post transfection and fresh medium was added to each plate and incubated for 24 h.

### Co-immunoprecipitation

Magna ChIP™ A/G Chromatin Immunoprecipitation kit (MilliporeSigma, Burlington, MA) was used to investigate APC and TRP120 interaction during *E. chaffeensis* infection at 0, 6 and 10 hpi. THP-1 cells were infected with *E. chaffeensis* (MOI 100) and harvested for Co-IP according to the manufacturer’s protocol. APC and TRP120 antibodies were used to determine interactions. IgG purified from normal serum was used as control antibody. Bound antigen was eluted, solubilized in 4X SDS sample loading buffer, and processed for immunoblot analysis. The membrane was probed with APC or TRP120 antibody to confirm pulldown. Co-immunoprecipitation was performed with reversal and in triplicates.

### Ubiquitination of native APC

Uninfected and *E. chaffeensis*-infected THP-1 cell lysates (800 µg) were subjected to ubiquitin pulldowns (Enzo Life Sciences, Farmingdale. NY, United States) using Ubiquitin Enrichment Kit (Pierce, Appleton, WI, United States) following the manufacturer’s protocol. Samples were boiled for 5 min and resolved by SDS-PAGE and processed for Western blotting using anti-APC (Abnova) (Figure 4A). APC ubiquitination was performed with purified native APC protein from THP-1 cells. TRP120-FL, TRP120-C520S and purified native APC protein were added to ubiquitination buffer in the presence of E1, ATP and UbcH5b (50 µL total volume) according to the manufacturer’s protocol. Ubiquitination reactions were performed at 37°C for 3 h and the reactions were stopped by addition of Laemmli buffer. Samples were boiled for 5 min and resolved by SDS-PAGE and processed for Western blotting using anti-APC and anti-Ub antibodies (Figure 4B).

### Statistical analysis

Statistical analysis involving multiple comparisons was performed using ANOVA and Tukey’s *post hoc* analysis. Pairwise comparisons of means were performed with a pairwise Student’s t-test.

## Discussion

*E. chaffeensis* exploits evolutionarily conserved host cell signaling pathways, including Hippo, Wnt, Notch and Hedgehog to prevent apoptosis and other innate immune defenses for intracellular survival (Rogan et al., 2021; Byerly et al., 2022; Patterson et al., 2022; Byerly et al., 2023a). Both Hippo and Wnt are known for their role in regulating cellular proliferation and cell fate (He et al., 2015; Wang et al., 2017; Qian et al., 2021), and we identified TRP120 as a novel Wnt ligand mimic that activates Yap and β-catenin, preventing apoptosis for ehrlichial survival (Rogan et al., 2021; Byerly et al., 2023a). Yap and β-catenin activate gene transcription under two conditions; when Hippo signaling is inactivated and APC is degraded (Cai et al., 2015; Park et al., 2015; Wang et al., 2017; Lee et al., 2018; Jiang et al., 2020). Recently, we determined that *E. chaffeensis* TRP120 moonlights as a HECT-type E3 ubiquitin ligase and targets PCGF5, FBW7 and ENO-1 in a proteosome dependent manner to exploit host cell mechanisms, including chromatin remodeling, cell signaling and glycolytic flux (Zhu et al., 2017; Mitra et al., 2018; Wang et al., 2020; Zhu and McBride, 2021). In this study, we identify APC as a novel substrate of TRP120, showcasing TRP120-mediated ubiquitination and temporal degradation of APC, thereby stabilizing active Yap and β-catenin levels in the nucleus. Since TRP120s E3 ubiquitin ligase activity targets PCGF5, FBW7, and ENO-1 for degradation in a proteasome-dependent manner, a similar mechanism appears to regulate APC.

APC was first discovered in 1991 for its involvement in familial adenomatous polyposis (FAP), which is an autosomal dominant inherited disorder characterized by the early onset of adenomatous polyps throughout the colon (Macpherson et al., 1992; Hryhorowicz et al., 2022). If left untreated, FAP progresses to colon cancer. The genetic defect that causes FAP is a germline mutation in *APC* (Ripa et al., 2002). APC is a chaperone protein that shuttles Yap and β-catenin out of the nucleus to prevent cell proliferation and tumorigenesis. However, mutations in *APC* lead to inadequate shuffling and accumulation of Yap and β-catenin in the nucleus (Henderson, 2000; Brocardo et al., 2005; Azzolin et al., 2014; Cai et al., 2015). Here, we reveal that reduced APC promotes ehrlichial survival and stabilizes active Yap and β-catenin in the nucleus in APC knockdown cells.

We determined that APC levels were significantly decreased during infection, despite upregulation of *APC* transcription, suggesting posttranscriptional regulation of APC, either by translational inhibition or proteasomal degradation. Notably, recently studies have shown that MKRN1, a host E3 ligase ubiquitinates APC for proteasomal degradation and mutations in MKRN1 E3 ligase domain impaired APC regulation, indicating that E3 Ub ligase activity is required for APC regulation. In addition, MKRN1 knockdown also resulted in reduced active β-catenin (Lee et al., 2018). Similarly, our studies reveal that TRP120 HECT E3 ligase directly interacts with and ubiquitinates APC to regulate APC levels. Using TRP120 HECT E3 ubiquitin ligase mutant (TRP120-C520S), we demonstrated that APC levels are stabilized, resulting in reduced levels of active Yap and β-catenin in the nucleus. In cells infected with *E. chaffeensis* combined with APC knockdown, we observed increased active Yap and β-catenin compared to *E. chaffeensis* infection alone (scRNA), indicating that enhanced reduction of APC through RNAi further increases stabilization of active Yap and β-catenin levels in the nucleus. The combination of *E. chaffeensis* infection with APC knockdown yielded the highest fold change in Yap and β-catenin levels which is likely a result of further reduction of APC over infection alone. The effect of APC knockdown on *E. chaffeensis* infection is consistent with other defined TRP120 substrates (PCGF5, FBW7, and ENO1) that are targeted for proteasomal degradation to promote ehrlichial infection.

TRP120 colocalization and interaction with APC was observed at 6 and 10 hpi. However, results differed when comparing full length (FL) and truncated (TR) APC. The co-immunoprecipitation assay demonstrated more interaction between TRP120 and APC-TR than APC-FL. This likely occurs due to APC levels within the cell. Both APC-FL and -TR reside within the cytoplasm and nucleus and have inhibitory effect on Yap and β-catenin. However, based on our results, there are higher amounts of detectable APC-TR than APC-FL in THP-1 cells. Regardless, total APC protein levels decrease temporally during infection, with no detectable total APC by 72 hpi, which was demonstrated via confocal microscopy and Western blot.

To demonstrate ubiquitination of APC, we used an enrichment assay to demonstrate that APC is ubiquitinated during early infection when infectious dense core ehrlichiae are present. However, ubiquitinated APC was undetectable during mid to late infection when *Ehrlichia* replication is occurring. One explanation for this observation, is that *E. chaffeensis* is targeting APC before replication begins, to prevent host-cell apoptosis and is consistent with previous findings that demonstrate *E. chaffeensis* activates Yap to prevent apoptosis (Byerly et al., 2023a). Collectively, these results demonstrate that TRP120 ubiquitinates APC to stabilize active Yap and β-catenin levels in the nucleus to promote infection.

**FIGURE 5:**
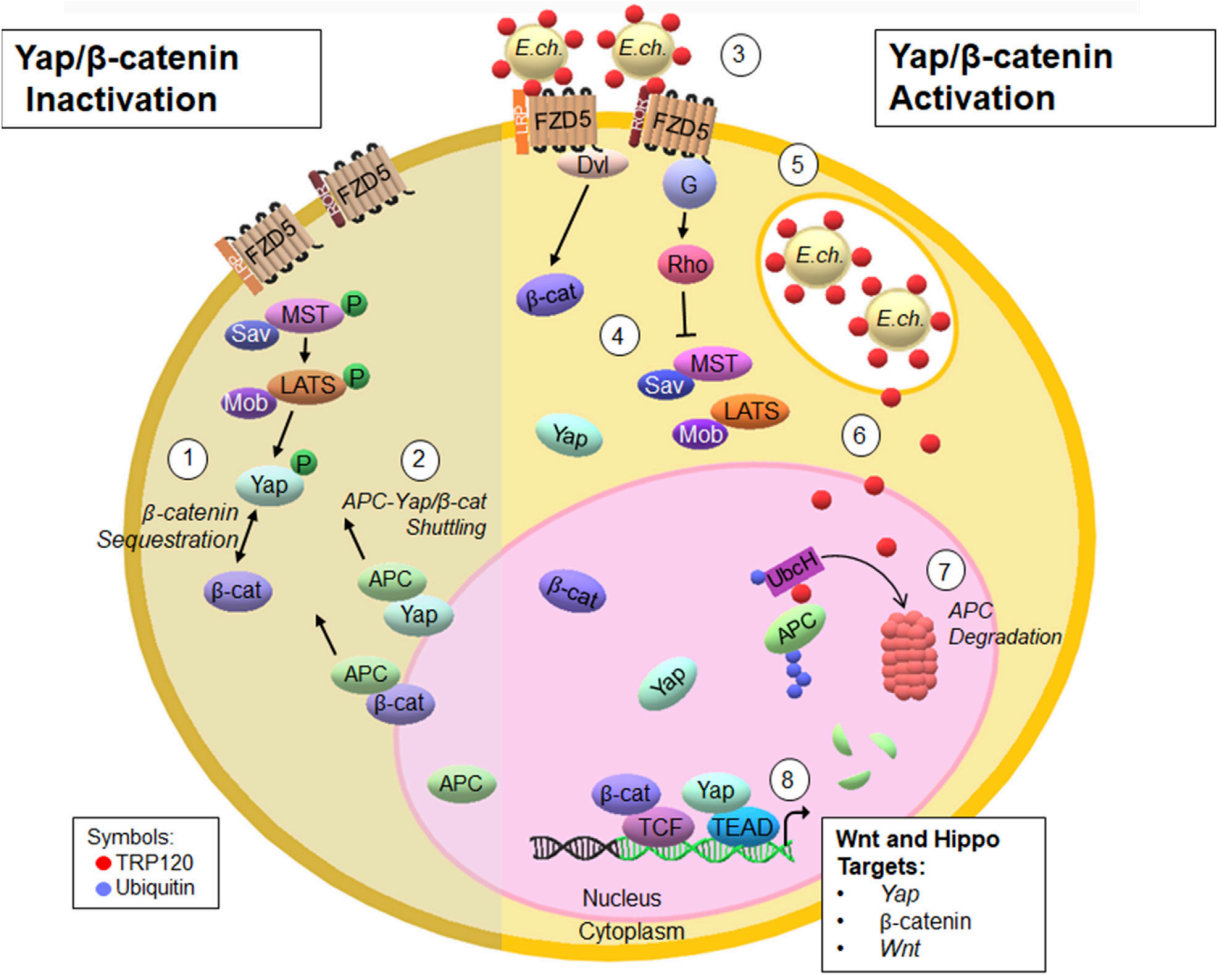
Proposed model of *E. chaffeensis* TRP120 HECT E3 ligase ubiquitination of APC to modulate Yap and β-catenin levels during infection (Paddock and Childs, 2003). The Hippo signaling cascade phosphorylates Yap and consequentially sequesters β-catenin within the cytoplasm (Paddock et al., 1997). APC shuttles Yap and β-catenin from the nucleus to the cytoplasm, preventing transcription (Rogan et al., 2021). *E. chaffeensis* secretes TRP120 via the T1SS to interact with Fzd5 (Byerly et al., 2022). Upon TRP120 interaction with Fzd5, Hippo signaling is inhibited and Yap and β-catenin translocate to the nucleus to activate transcription (Patterson et al., 2022). Infectious dense-cored *E. chaffeensis* expressing TRP120 on the surface enter host monocytes through receptor-mediated phagocytosis and resides within cytoplasmic vacuoles that do not fuse with lysosomes (Byerly et al., 2023a); TRP120 is secreted via the T1SS from the vacuole to the nucleus (Wakeel et al., 2011); TRP120 binds and ubiquitinates APC. APC- Ub is then degraded by the proteasome, presumably in the nucleus (Wakeel et al., 2009). TRP120-mediated degradation of APC leads to Yap and β-catenin activation and regulation of downstream gene targets.

Studies that have investigated pathogen manipulation of APC are scarce. Case studies have identified novel frameshift mutations in *APC*, which leave individuals susceptible to gastric cancer after *Helicobacter pylori* infection (Mitsui et al., 2019). Additionally, DNA methylation of *APC* in patients with human papillomavirus, puts them at high risk for developing cervical cancer (Mao et al., 1996). Although mutations in *APC* are correlated to developing cancers after infection, a direct mechanism involving pathogen regulation of APC has never been described. This study is the first report of pathogen-directed manipulation of APC to modulate Hippo and Wnt signaling. In this study, we have unveiled a mechanism whereby an *Ehrlichia* ubiquitinates APC to coordinate with upstream signaling pathway activation to promote infection (Figure 5). This important finding not only reveals a new substrate and pathway targeted pathogen Ub ligase activity, but also holds immense potential for the development of innovative therapeutic approaches for pathogens that may exploit APC for infection. Furthermore, this study identifies a valuable model for understanding how pathogens can target APC to modulate infection.

## Data Availability

The original contributions presented in the study are included in the article/Supplementary Material, further inquiries can be directed to the corresponding author.
