# Supplementary figures and images for: Ehrlichia chaffeensis TRP120 ubiquitinates tumor suppressor APC to modulate Hippo and Wnt signaling
Source: Front Cell Dev Biol. 2024 Mar 18;12:1327418. doi: 10.3389/fcell.2024.1327418 (PMC10982408; doi:10.3389/fcell.2024.1327418)

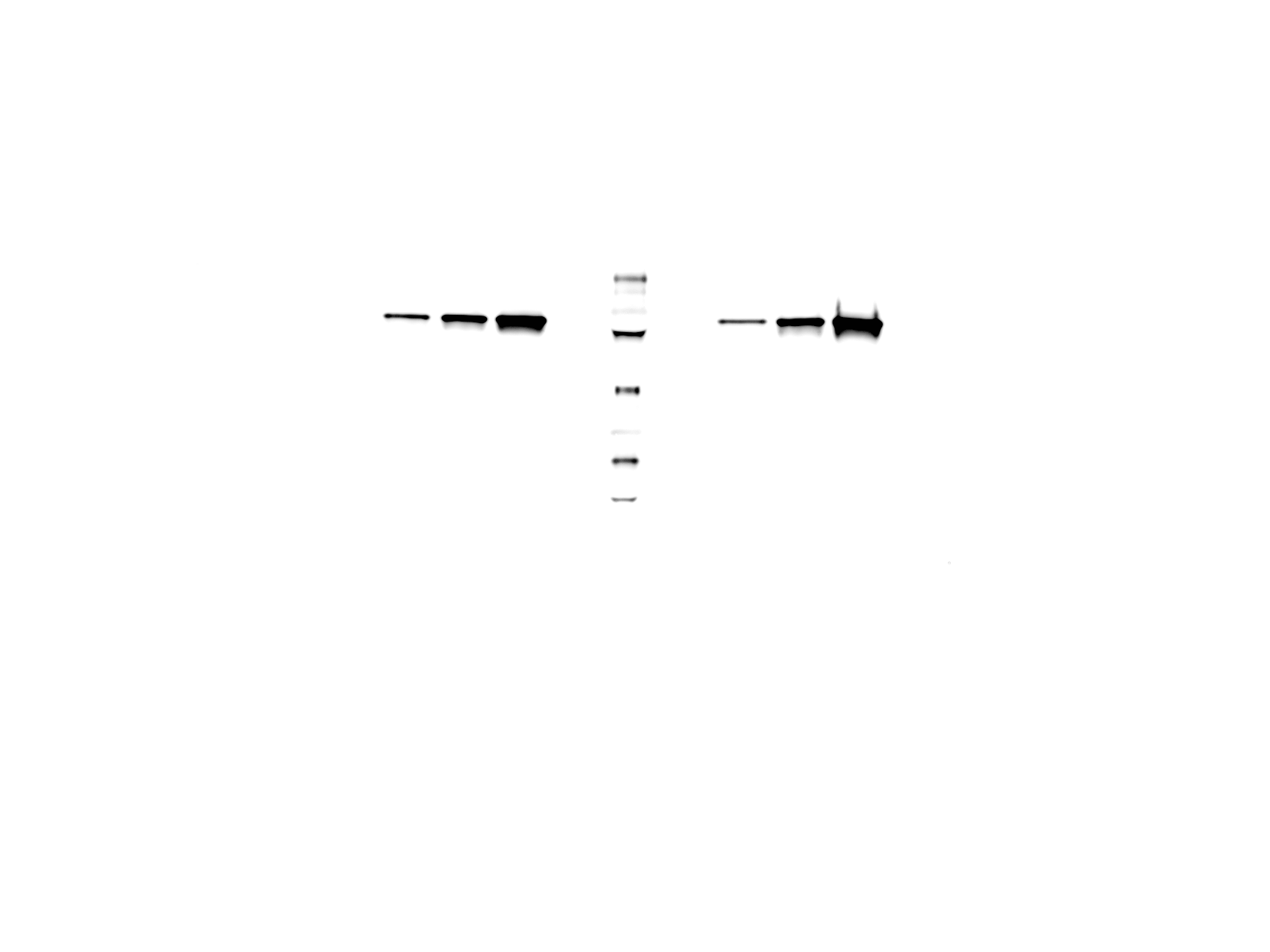

Supplement: Supplementary file 1 [file DataSheet1.ZIP › Fig 1-C DSB.TIF]

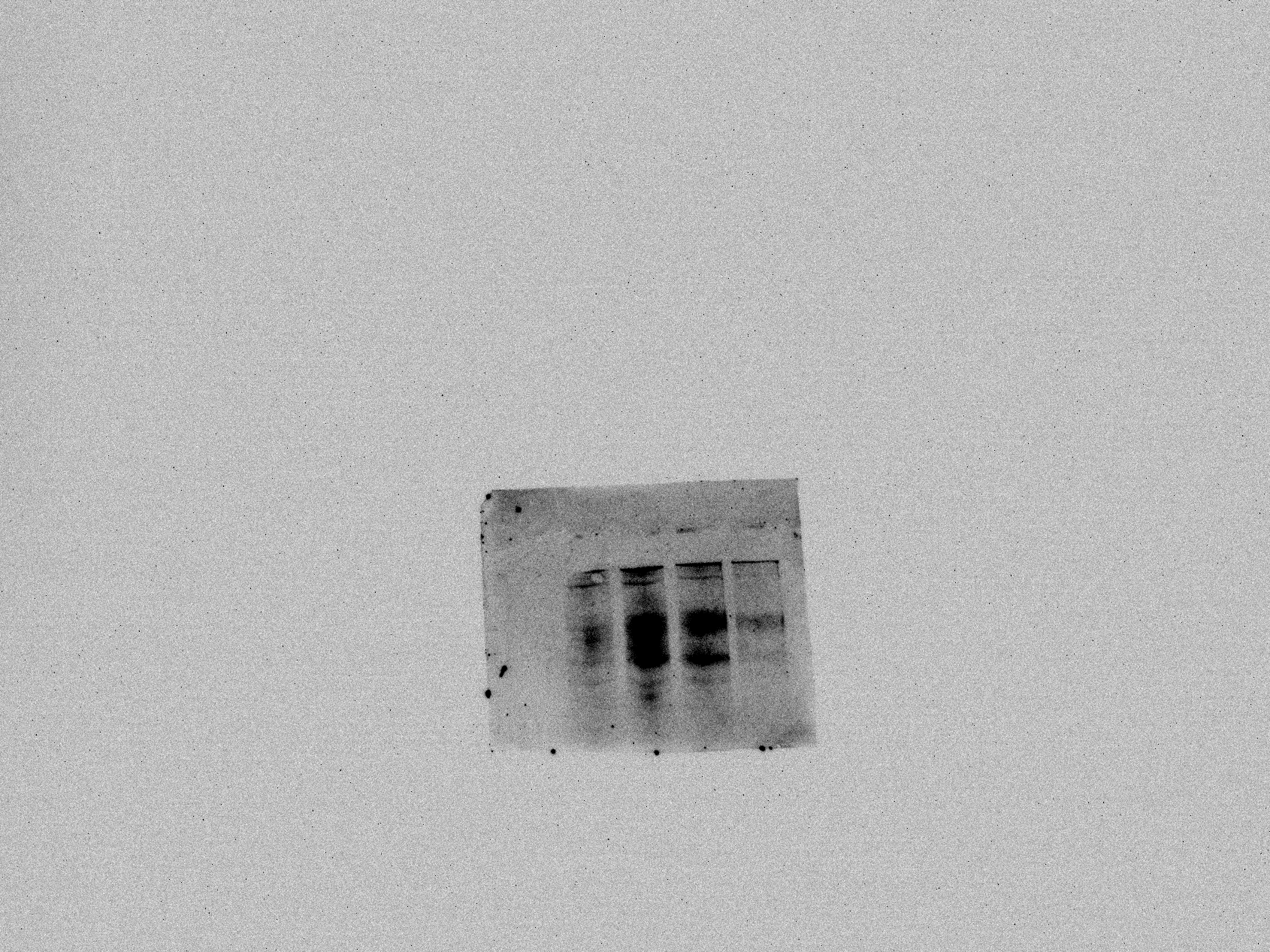

Supplement: Supplementary file 1 [file DataSheet1.ZIP › Fig 1-C-APC-FL.tif]

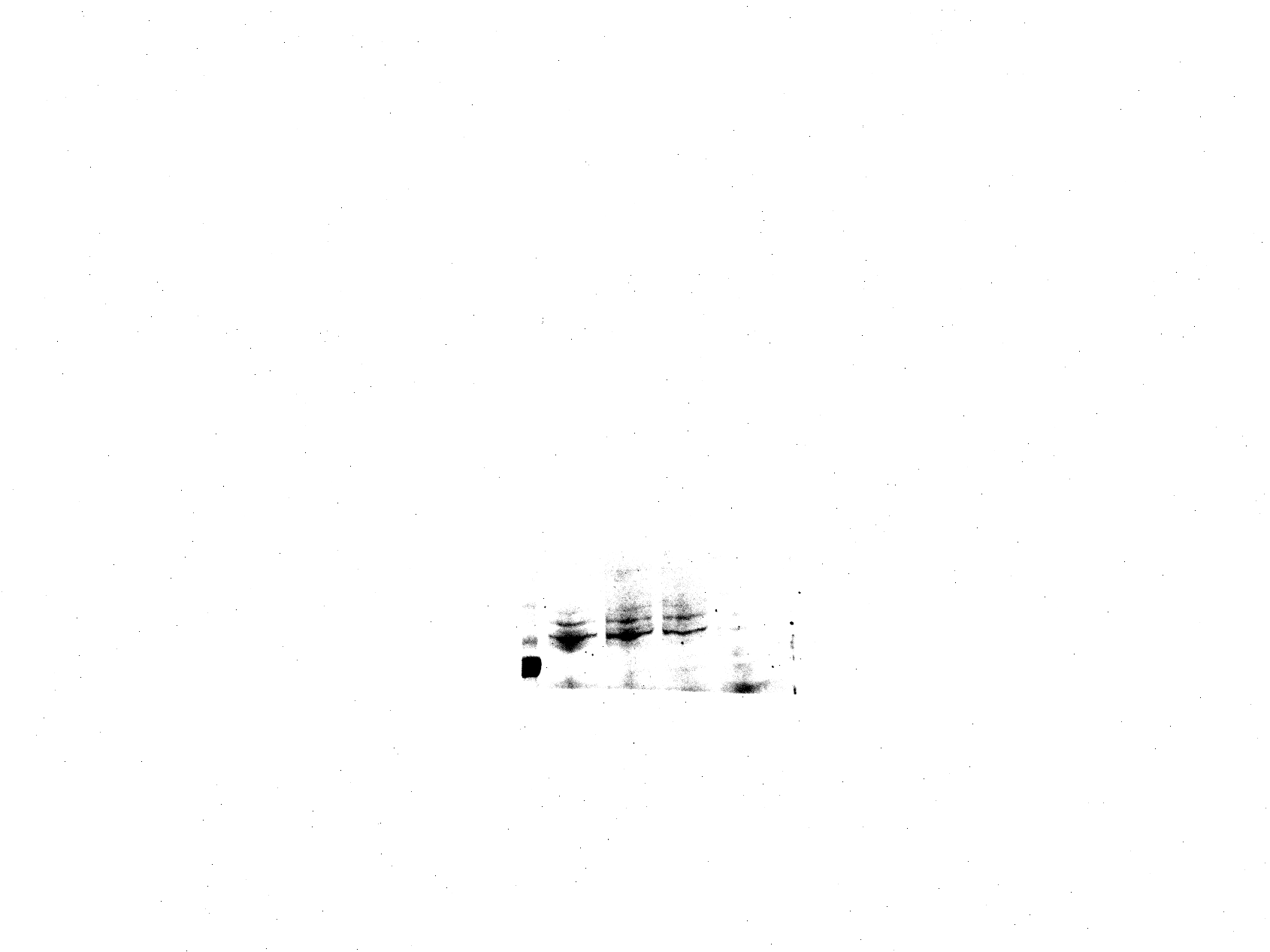

Supplement: Supplementary file 1 [file DataSheet1.ZIP › Fig 1-C-APC-TR.TIF]

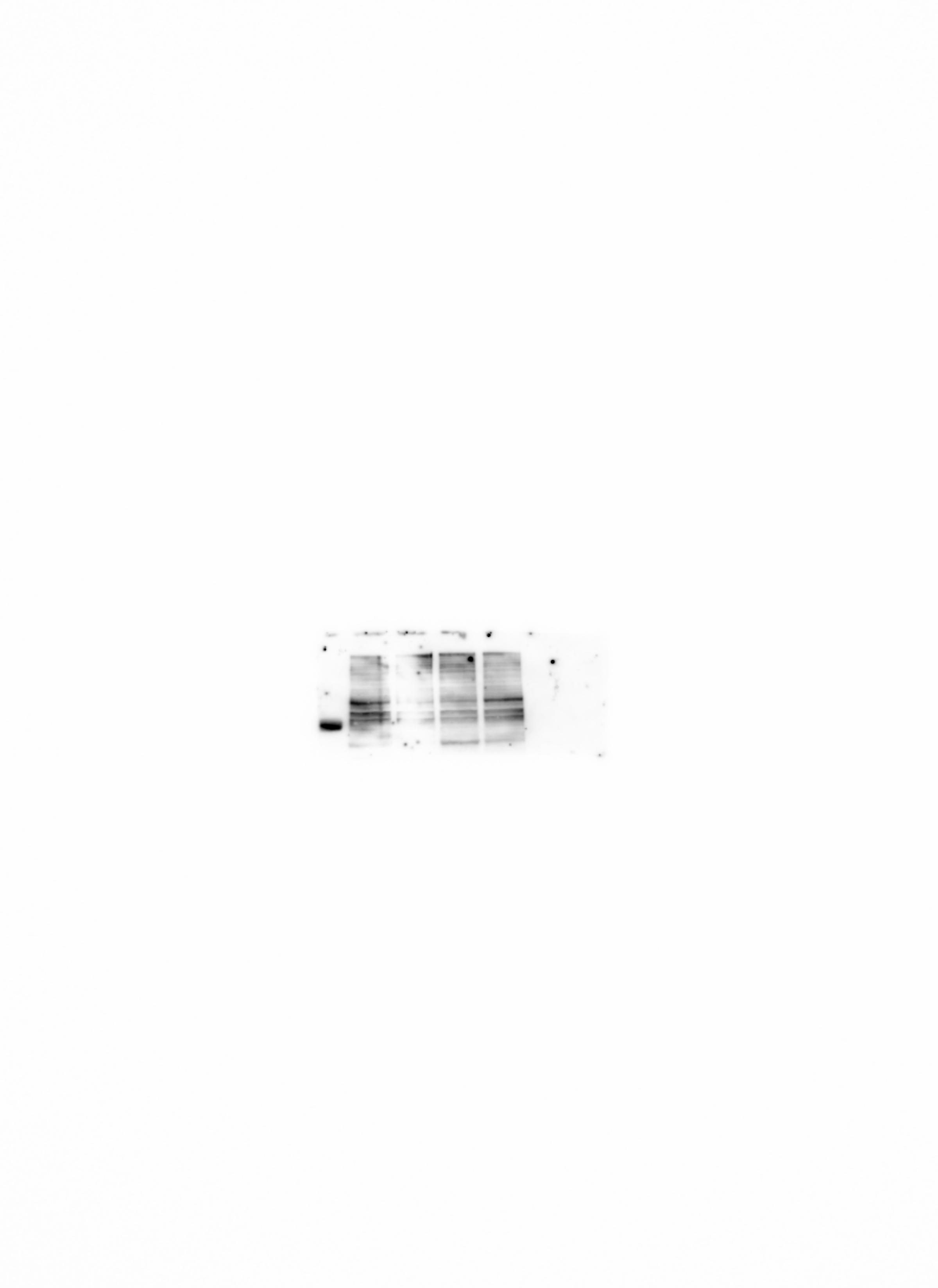

Supplement: Supplementary file 1 [file DataSheet1.ZIP › Fig 2-A apc kd.tif]

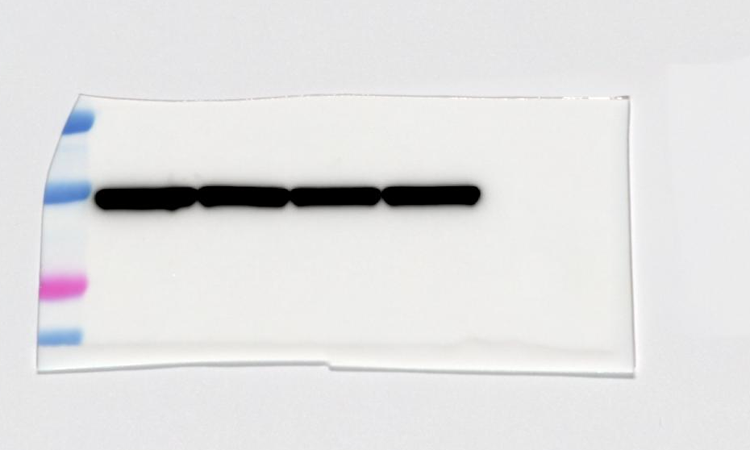

Supplement: Supplementary file 1 [file DataSheet1.ZIP › Fig 2-A GAPDH copy.tif]

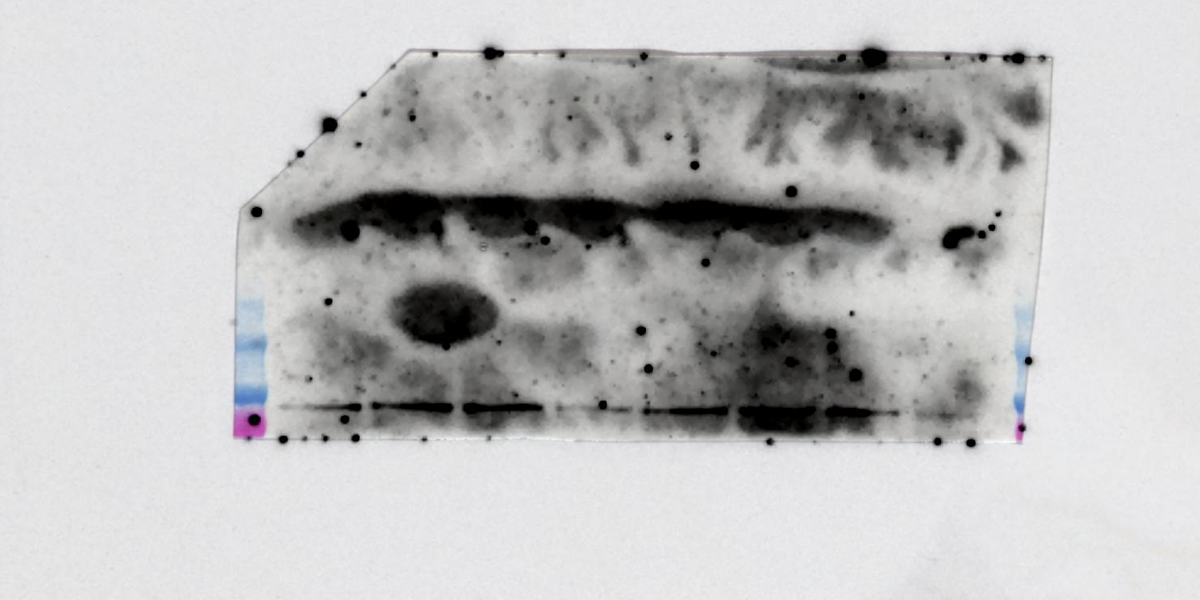

Supplement: Supplementary file 1 [file DataSheet1.ZIP › Fig 2-B b-cat copy.tif]

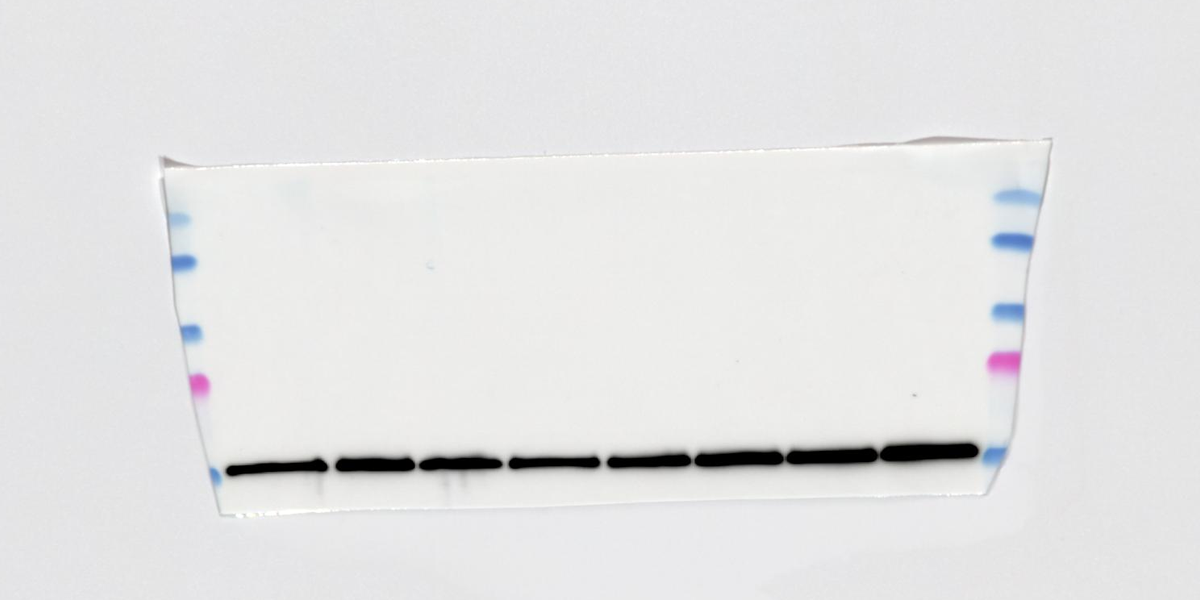

Supplement: Supplementary file 1 [file DataSheet1.ZIP › Fig 2-B Gapdh copy.tif]

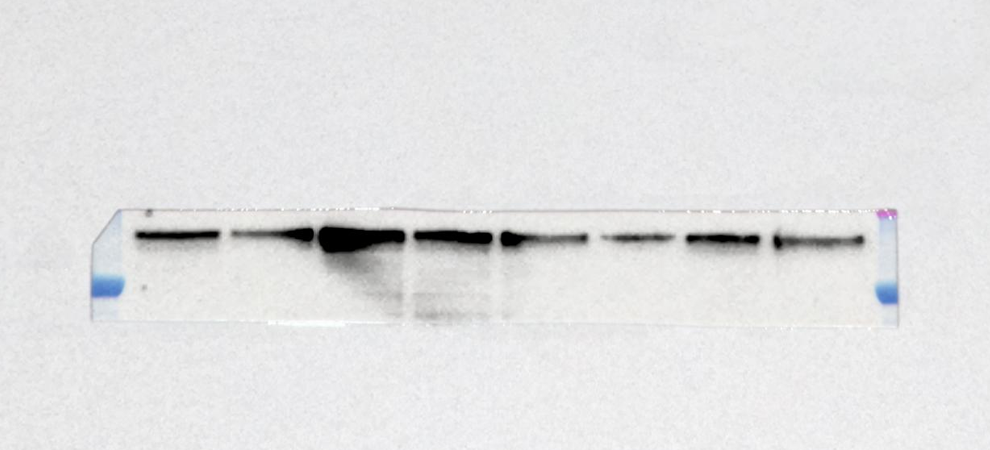

Supplement: Supplementary file 1 [file DataSheet1.ZIP › Fig 2-C Yap copy.tif]

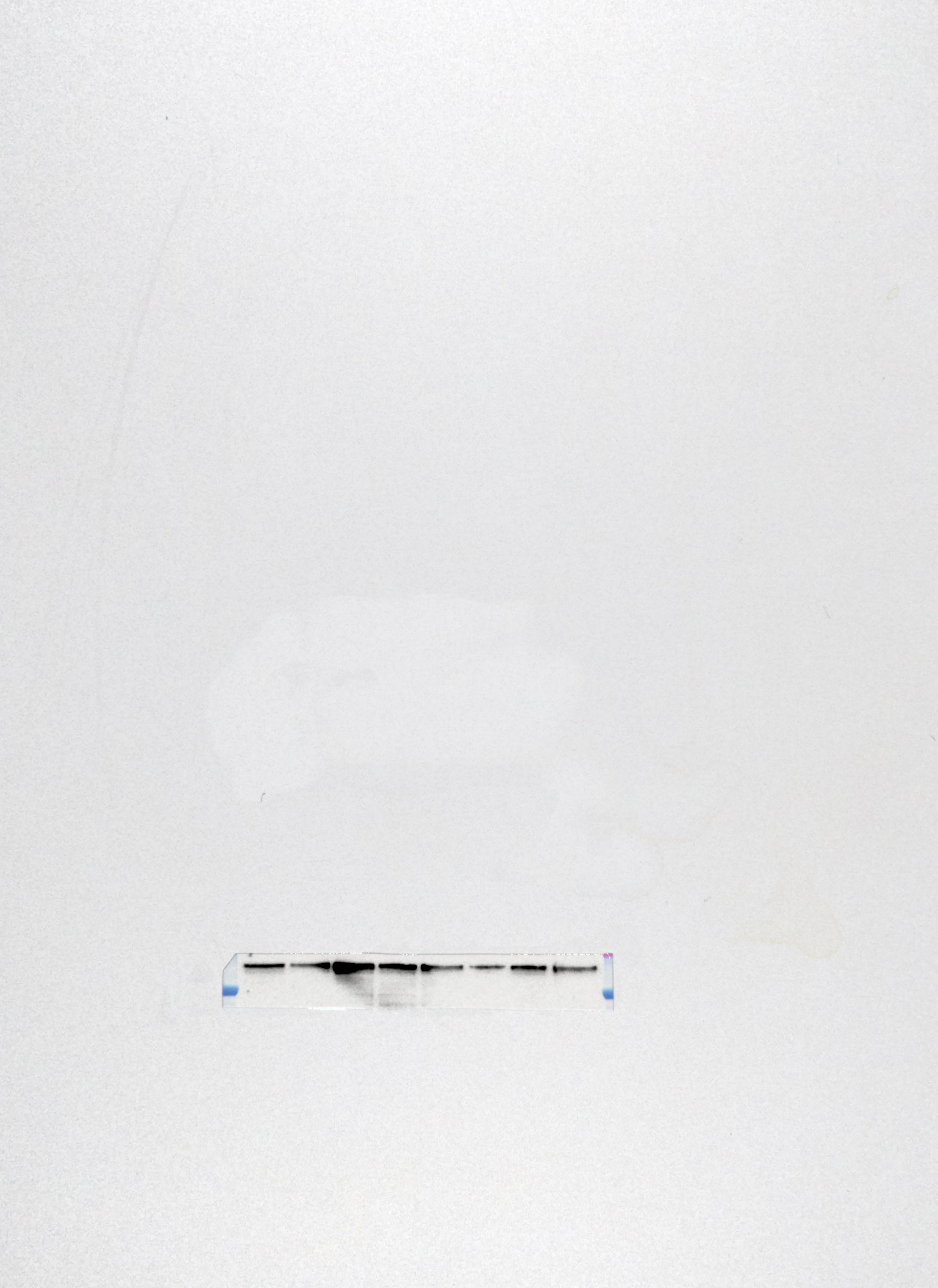

Supplement: Supplementary file 1 [file DataSheet1.ZIP › Fig 2-C Yap.tif]

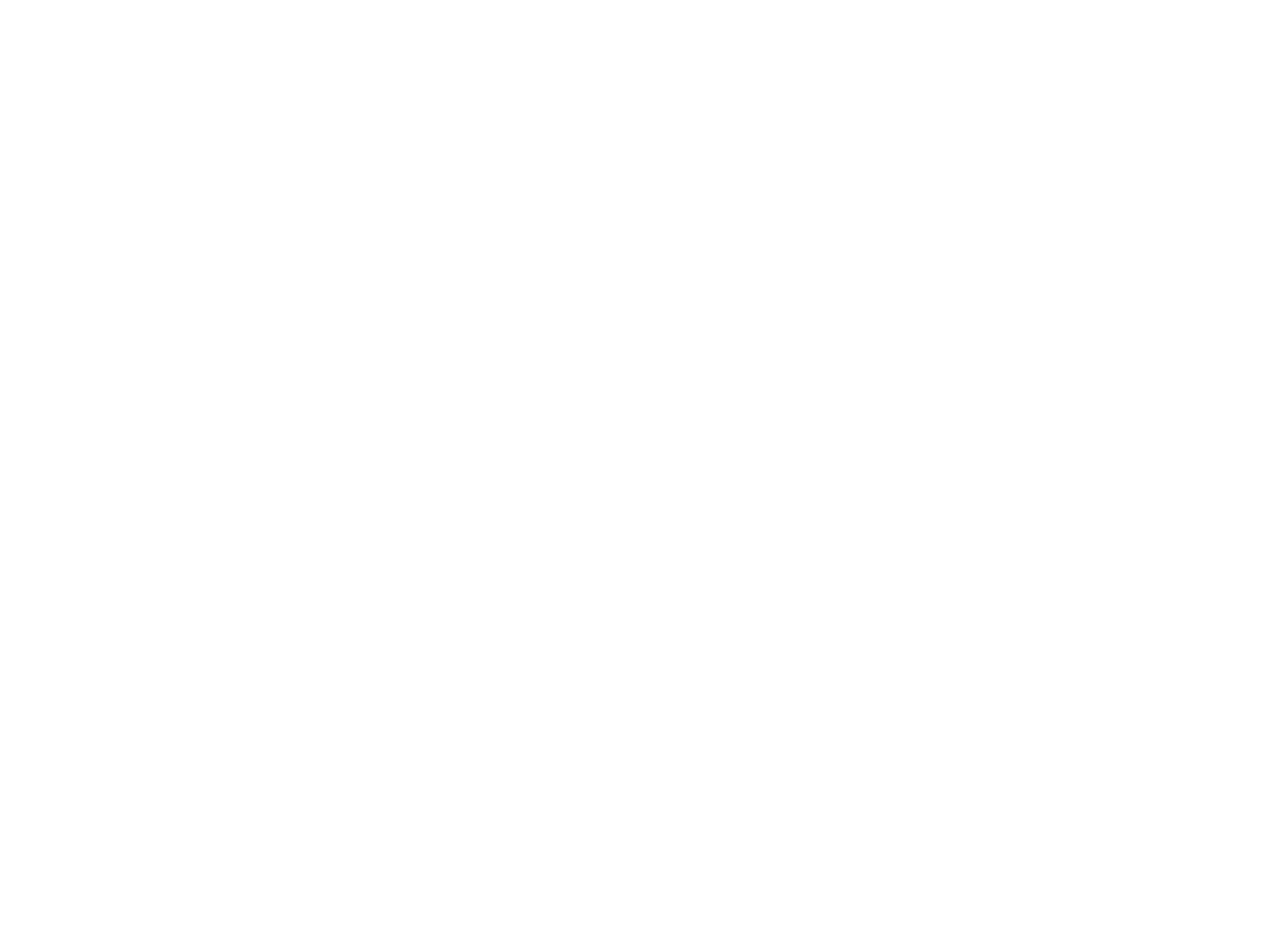

Supplement: Supplementary file 1 [file DataSheet1.ZIP › Fig 3 anti igg cont 1.tif]

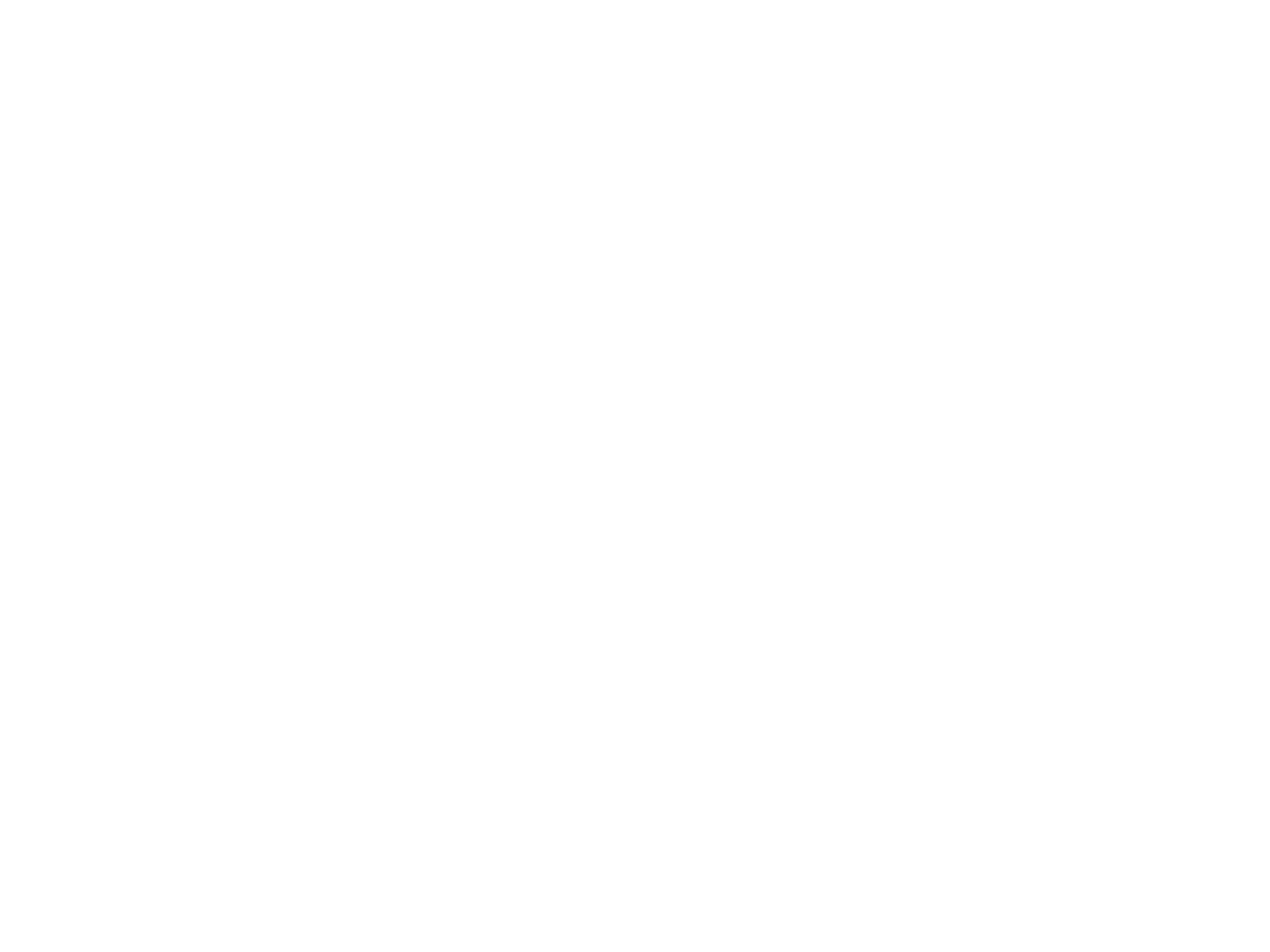

Supplement: Supplementary file 1 [file DataSheet1.ZIP › Fig 3 anti igg cont 2.tif]

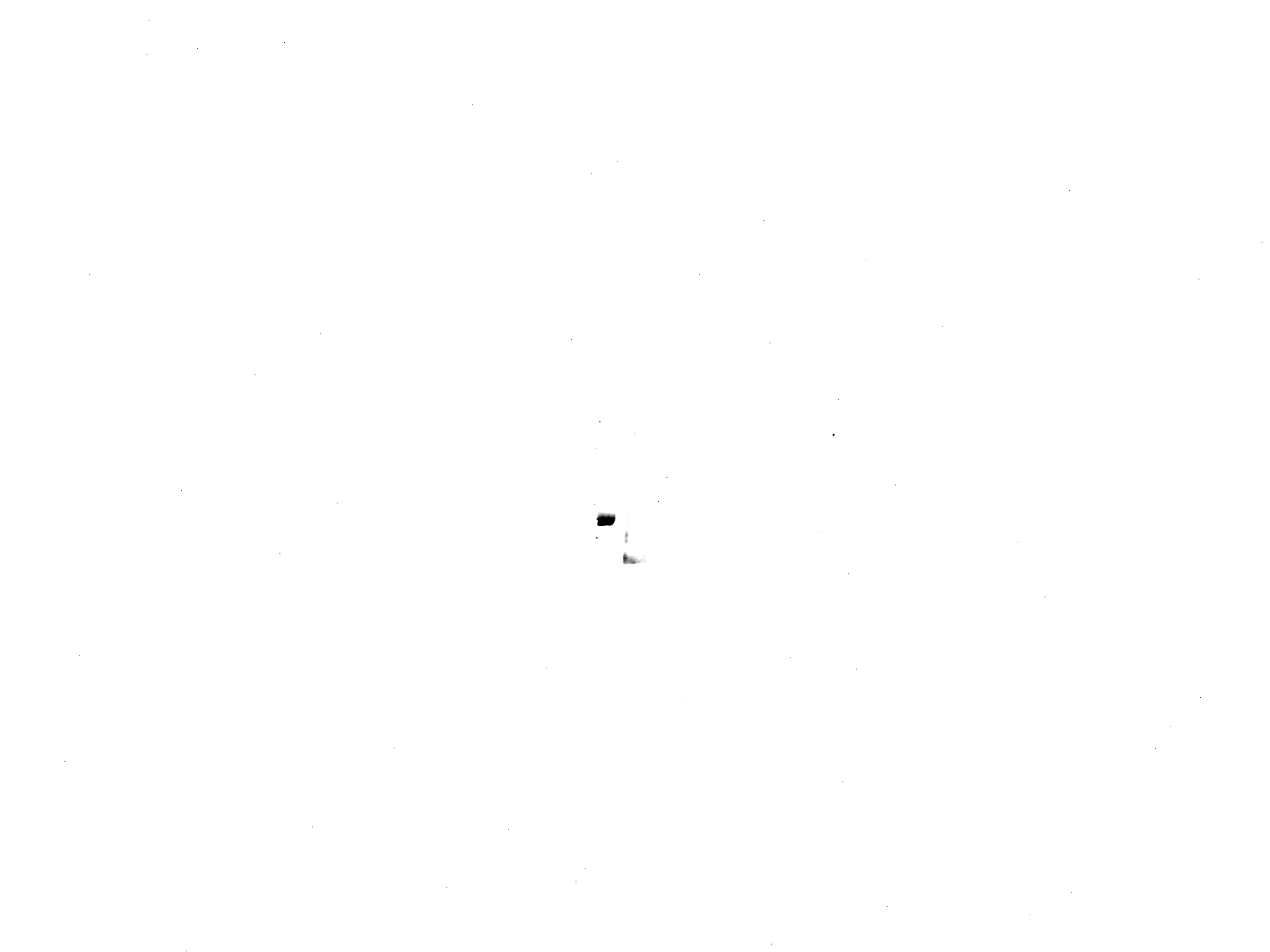

Supplement: Supplementary file 1 [file DataSheet1.ZIP › Fig 3 IgG cont.TIF]

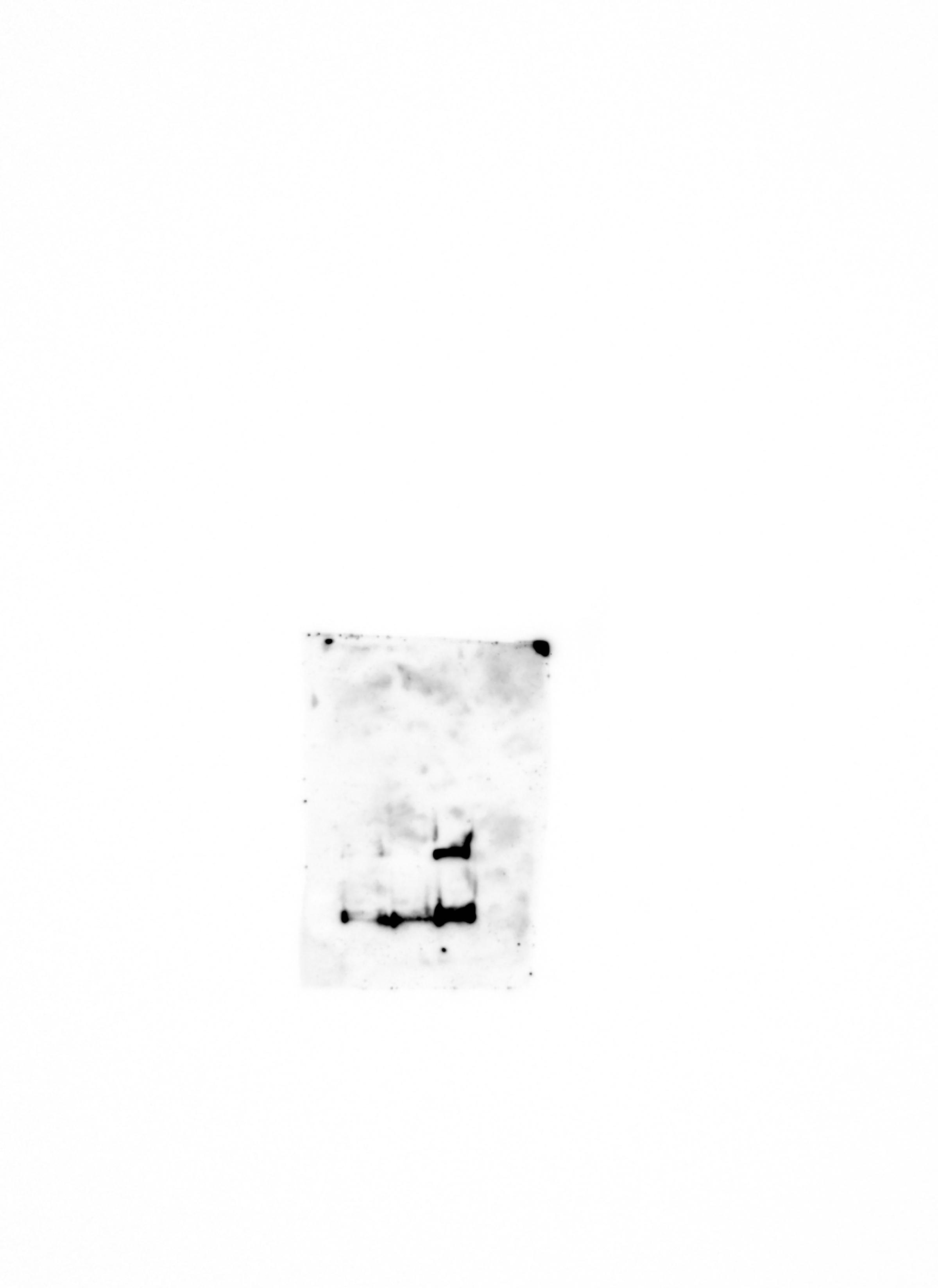

Supplement: Supplementary file 1 [file DataSheet1.ZIP › Fig 3-D-anti-APC.tif]

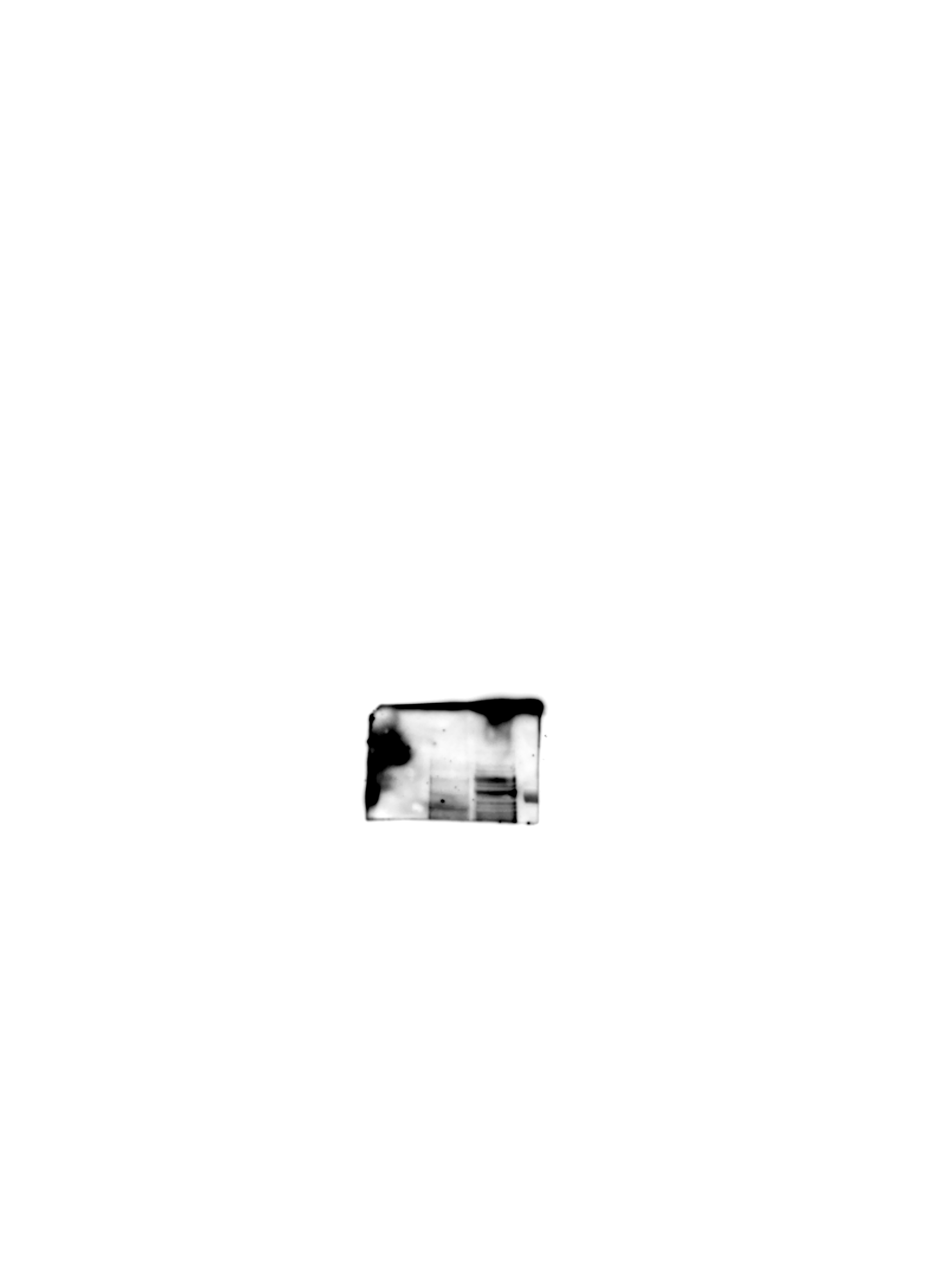

Supplement: Supplementary file 1 [file DataSheet1.ZIP › Fig 3-D-anti-APC-FL.tif]

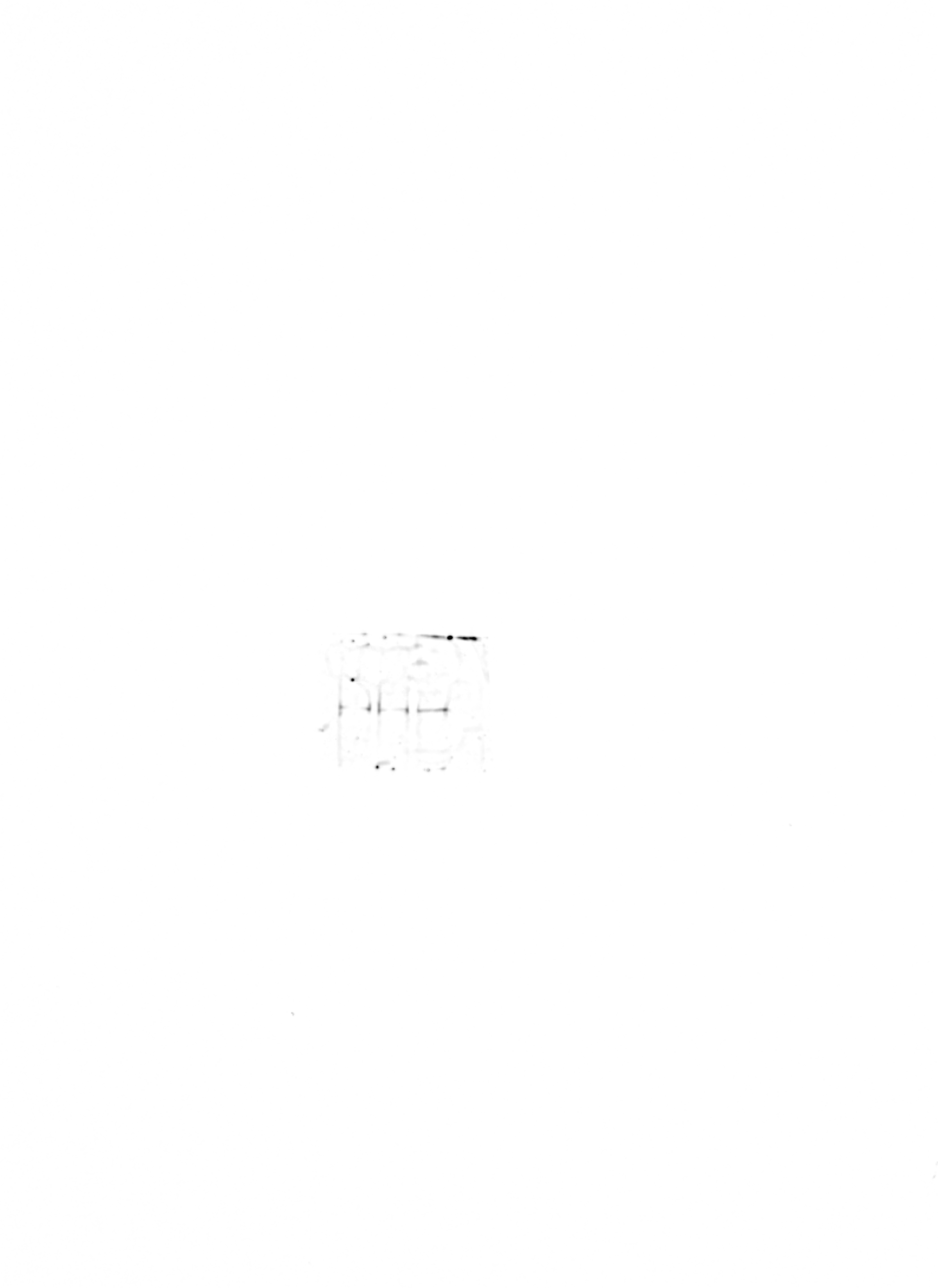

Supplement: Supplementary file 1 [file DataSheet1.ZIP › Fig 3-D-anti-TRP120.tif]

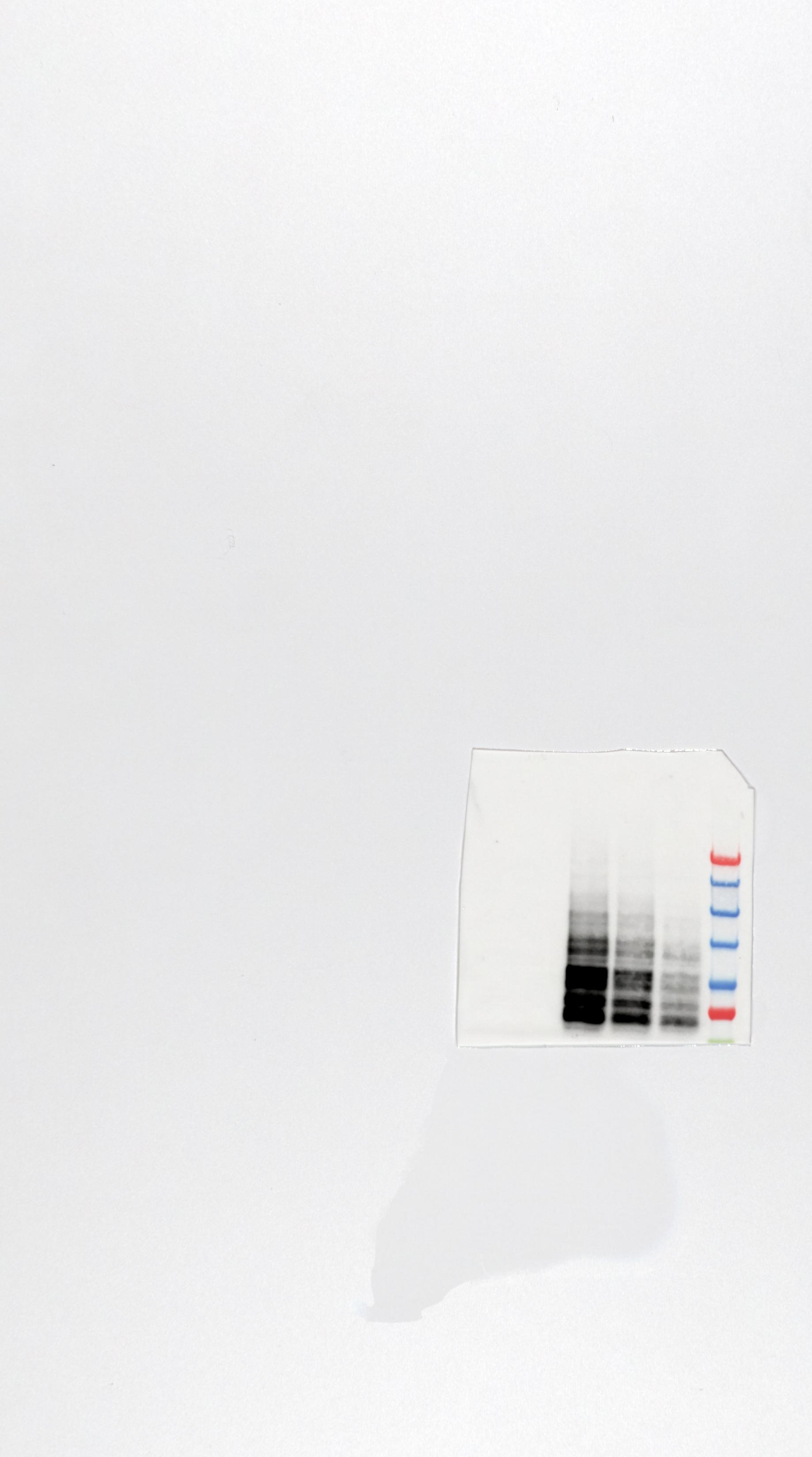

Supplement: Supplementary file 1 [file DataSheet1.ZIP › Fig 3-D-APC-input.tif]

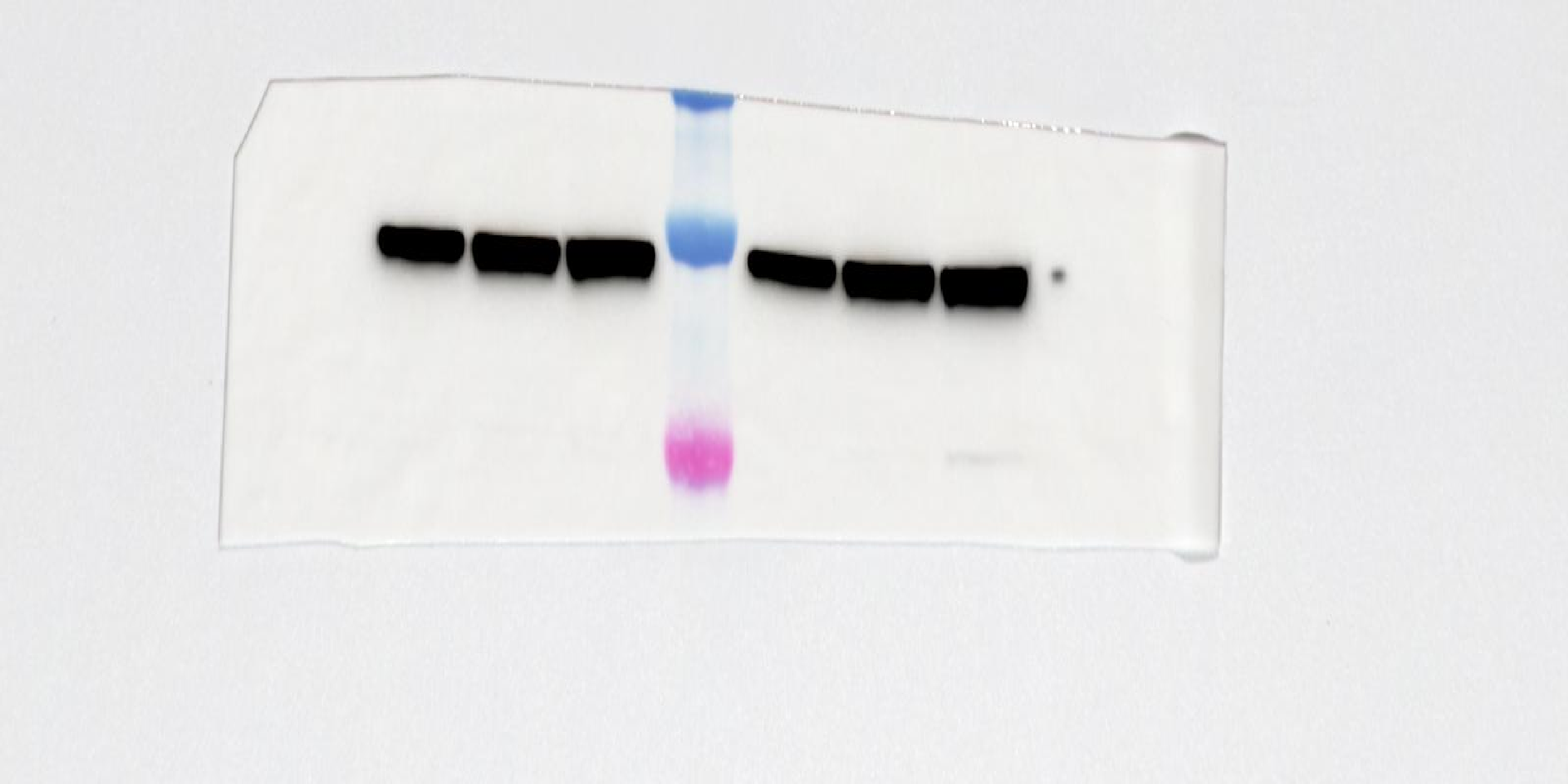

Supplement: Supplementary file 1 [file DataSheet1.ZIP › Fig 3-D-GAPDH-input copy.tif]

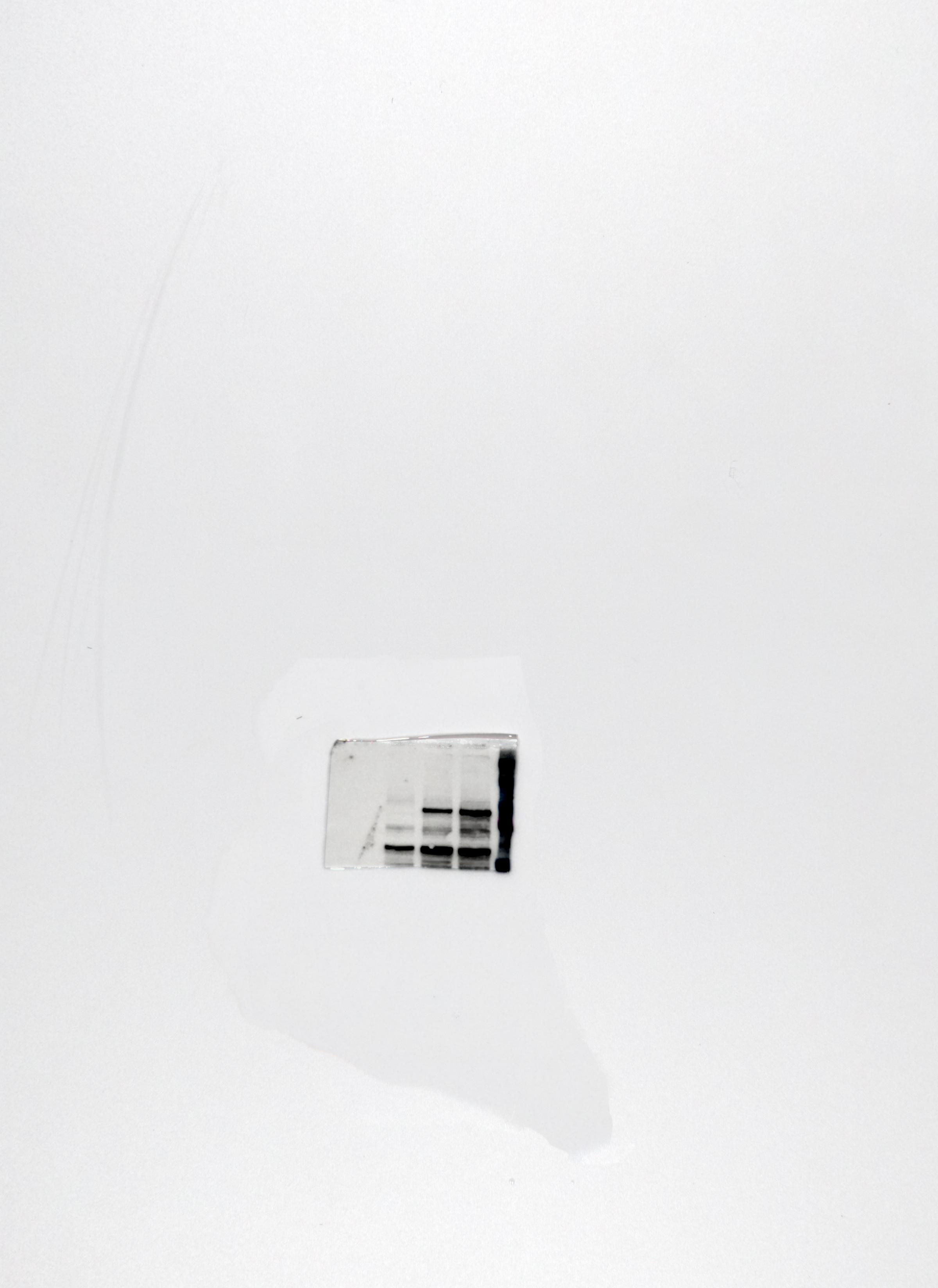

Supplement: Supplementary file 1 [file DataSheet1.ZIP › Fig 3-D-IgG-input.tiff]

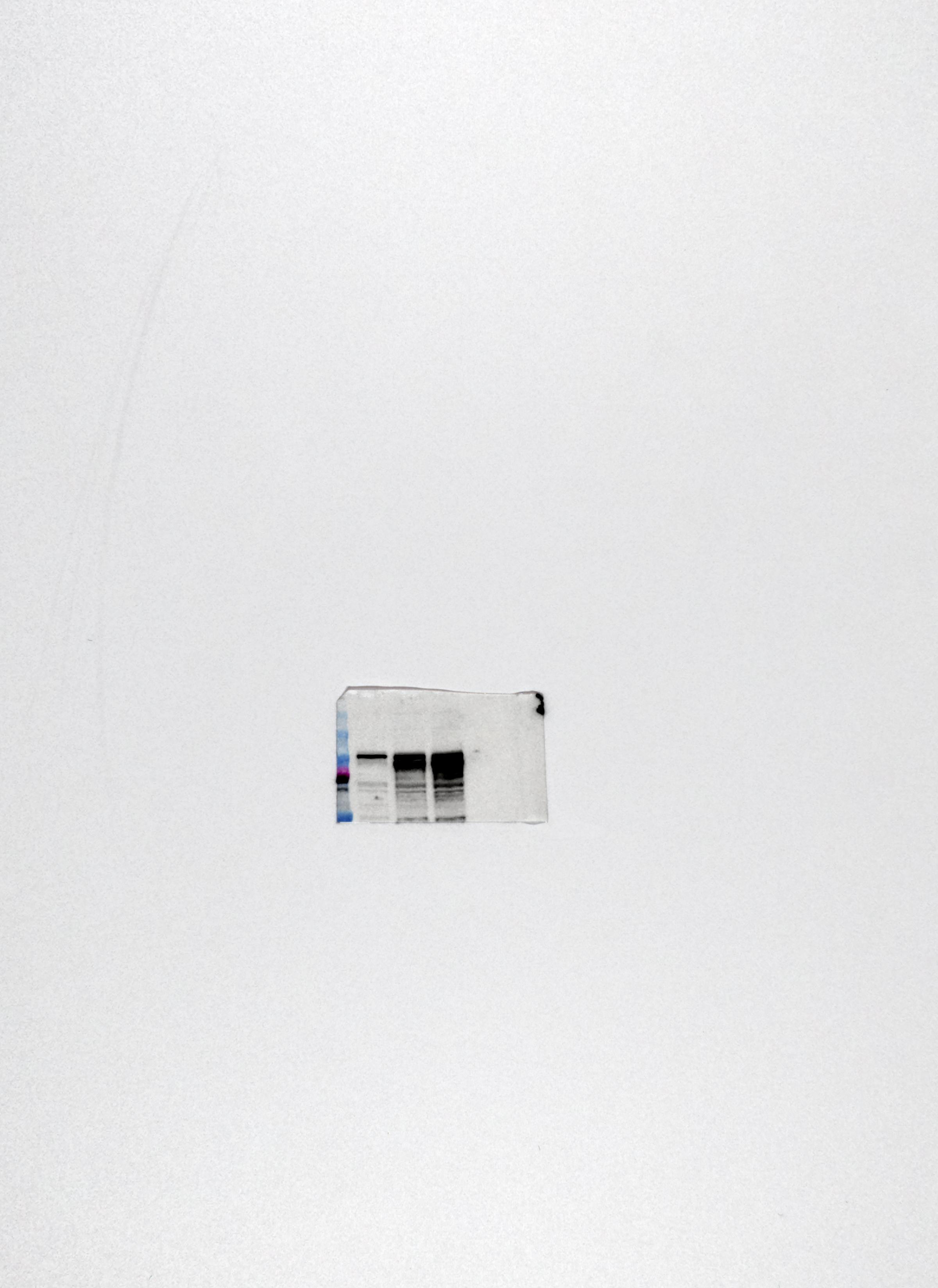

Supplement: Supplementary file 1 [file DataSheet1.ZIP › Fig 3-D-TRP-input.tif]

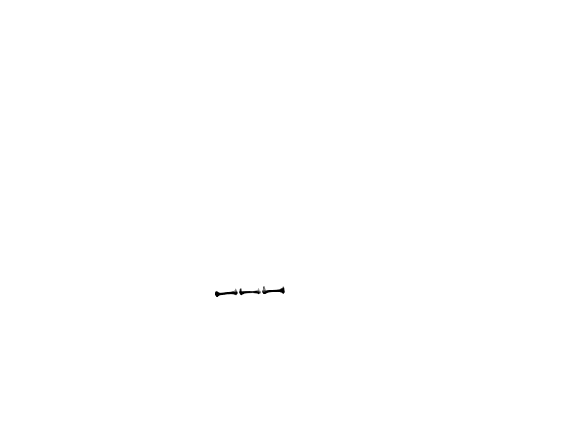

Supplement: Supplementary file 1 [file DataSheet1.ZIP › Fig 4-C-a-actinin.tif]

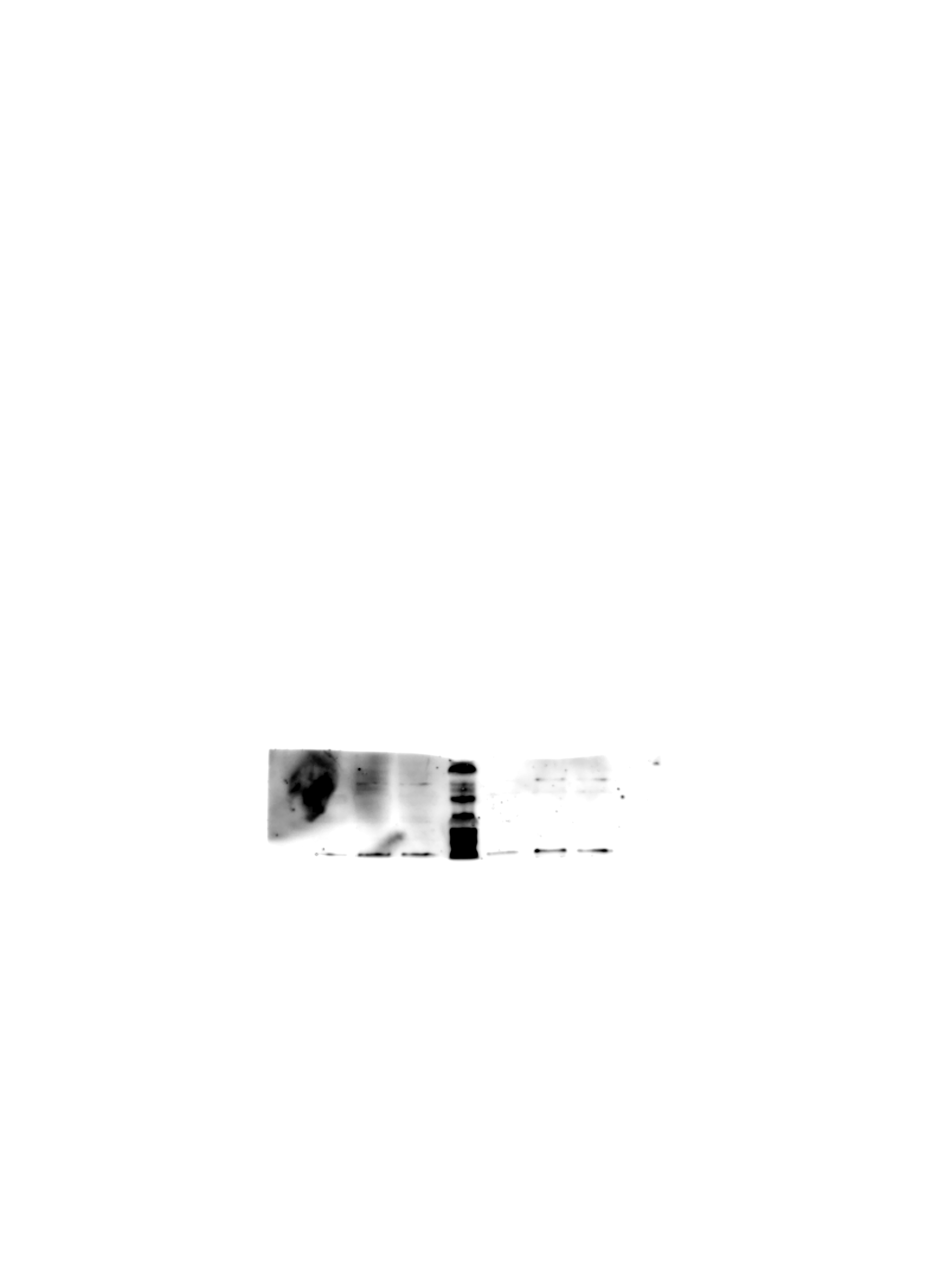

Supplement: Supplementary file 1 [file DataSheet1.ZIP › Fig 4-C-Active b-cat.tif]

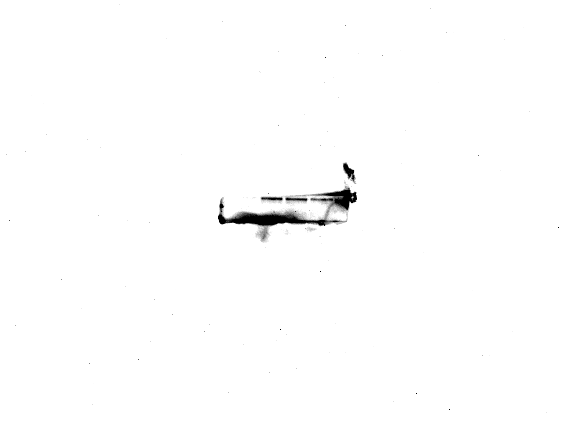

Supplement: Supplementary file 1 [file DataSheet1.ZIP › Fig 4-C-Active Yap.tif]

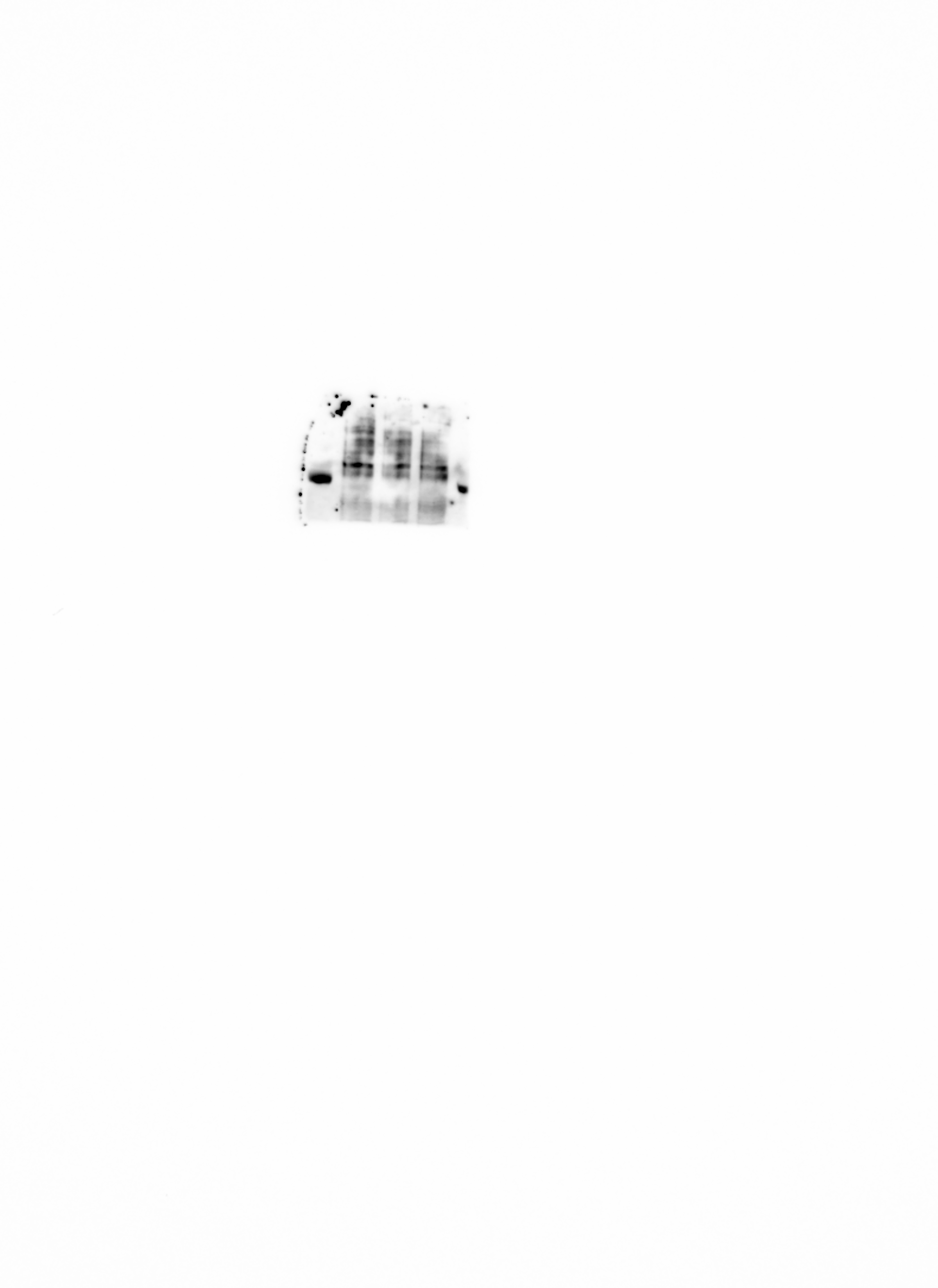

Supplement: Supplementary file 1 [file DataSheet1.ZIP › Fig 4-C-APC-FL.tif]

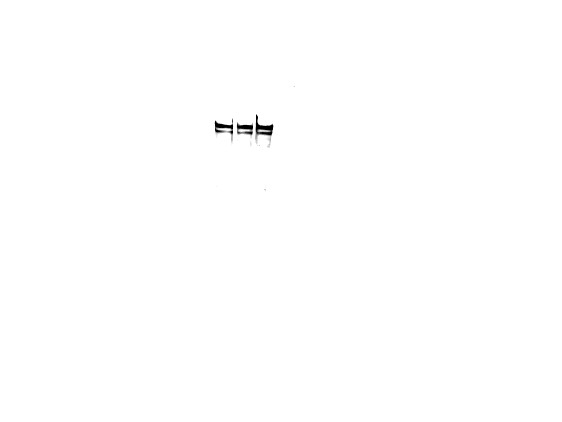

Supplement: Supplementary file 1 [file DataSheet1.ZIP › Fig 4-C-APC-Tr.TIF]

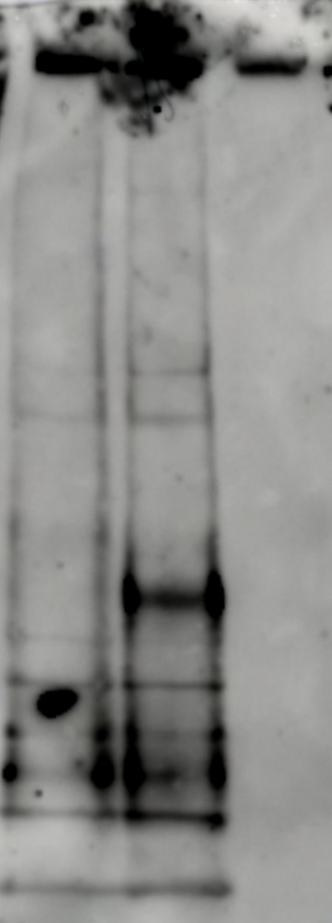

Supplement: Supplementary file 1 [file DataSheet1.ZIP › Fig4 A.TIF]

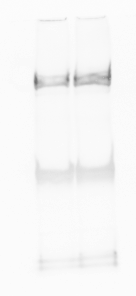

Supplement: Supplementary file 1 [file DataSheet1.ZIP › Fig4 B bottom panel.TIF]

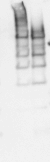

Supplement: Supplementary file 1 [file DataSheet1.ZIP › Fig4 B top panel.TIF]
